# Nrf2 Activation Mediates Antiallodynic Effect of Electroacupuncture on a Rat Model of Complex Regional Pain Syndrome Type-I through Reducing Local Oxidative Stress and Inflammation

**DOI:** 10.1155/2022/8035109

**Published:** 2022-02-14

**Authors:** Xiaojie Li, Chengyu Yin, Qimiao Hu, Jie Wang, Huimin Nie, Boyu Liu, Yan Tai, Junfan Fang, Junying Du, Xiaomei Shao, Jianqiao Fang, Boyi Liu

**Affiliations:** ^1^Department of Neurobiology and Acupuncture Research, The Third Clinical Medical College, Zhejiang Chinese Medical University, Key Laboratory of Acupuncture and Neurology of Zhejiang Province, Hangzhou 310053, China; ^2^Academy of Chinese Medical Sciences, Zhejiang Chinese Medical University, Hangzhou 310053, China

## Abstract

Complex regional pain syndrome type-I (CRPS-I) represents a type of neurovascular condition featured by severe pain in affected extremities. Few treatments have proven effective for CRPS-I. Electroacupuncture (EA) is an effective therapy for pain relief. We explored the mechanism through which EA ameliorates pain in a rat CRPS-I model. The chronic postischemic pain (CPIP) model was established using Sprague-Dawley rats to mimic CRPS-I. We found that oxidative stress-related biological process was among the predominant biological processes in affected hindpaw of CPIP rats. Oxidative stress occurred primarily in local hindpaw but not in the spinal cord or serum of model rats. Antioxidant N-acetyl cysteine (NAC) attenuated mechanical allodynia and spinal glia overactivation in CPIP model rats, whereas locally increasing oxidative stress is sufficient to induce chronic pain and spinal glia overactivation in naive rats. EA exerted remarkable antiallodynia on CPIP rats by reducing local oxidative stress via enhancing nuclear factor erythroid 2-related factor 2 (Nrf2) expression. Pharmacological blocking Nrf2 abolished antioxidative and antiallodynic effects of EA. EA reduced spinal glia overactivation, attenuated the upregulation of inflammatory cytokines, reduced the enhanced TRPA1 channel activity in dorsal root ganglion neurons innervating the hindpaws, and improved blood flow dysfunction in hindpaws of CPIP rats, all of which were mimicked by NAC treatment. Thus, we identified local oxidative injury as an important contributor to pathogenesis of animal CRPS-I model. EA targets local oxidative injury by enhancing endogenous Nrf2-mediated antioxidative mechanism to relieve pain and inflammation. Our study indicates EA can be an alternative option for CRPS-I management.

## 1. Introduction

Complex regional pain syndrome type-I (CRPS-I) is a debilitating pain condition that usually affects the extremities of the patients [1]. It can be triggered by an initial injury, including surgery, ischemia, and fracture, and can progress into a chronic stage that significantly affects the patients' daily activity [2, 3]. Clinical manifestations of CRPS-I include spontaneous pain, thermal/mechanical pain hypersensitivities, limb edema, microvascular injury, and blood flow abnormality [4]. Pain is among the clinical manifestations that mostly affect the patients, both physically and mentally [5, 6].

To study CRPS-I mechanisms, a rat chronic postischemic pain (CPIP) model was developed by applying prolonged ischemia, followed with reperfusion to the hind limb [7]. The CPIP model exhibited a number of key features that mimic clinical symptoms of CRPS-I, such as chronic thermal, mechanical, and chemical pain hypersensitivities in affected hind limbs, followed with microvascular injury and abnormalities in regional blood flow [8, 9]. The rat CPIP model has been widely applied for mechanistic studies of CRPS-I. Recently, we and others contributed to this field by identifying a number of pivotal mechanisms contributing to CRPS-I pathogenesis, including peripheral NMDA and TRPA1/TRPV1 channels, the chemokine CXCL12/CXCR4 signaling, NLRP3 inflammasome, and CSF1 in the spinal cord [10–14].

Peripheral tissues are densely innervated by free nerve endings that are projected from peripheral sensory ganglion (e.g., dorsal root ganglion). These free nerve endings act as nociceptors to detect neuroactive substances released from inflammatory cells or injured tissues, which trigger pain and/or produce pain sensitization. CRPS-I is related with microcirculation impairment associated with local tissue inflammation in the affected limb [15]. Therefore, pathophysiological changes in local tissue may play important roles in mediating the pathogenesis of CRPS-I.

Clinical trials failed to prove the beneficial effects of a number of conventional interventions, which makes CRPS-I a difficult-to-treat condition in the clinic [16, 17]. Therefore, there is an urgent need for alternative therapeutic options for CRPS-I. One promising alternative therapeutic option for CRPS-I is electroacupuncture (EA). EA combines traditional manual acupuncture with modern electrotherapy and has been widely used for relieving many kinds of pain conditions. Although the efficacy and safety of EA in relieving CRPS-I in clinic have been summarized recently [18], the detailed mechanisms underlying EA's therapeutic effects on CRPS-I still remain largely unexplored.

In this study, in order to explore the mechanisms underlying CRPS-I, we performed an unbiased RNA-Seq in local hindpaw tissues to explore potential substances or biological processes that are affected during CRPS-I. We then explored the potential involvement of these substances or processes in mediating CRPS-I and further examined whether EA exerts therapeutic effects on CRPS-I through acting on these mechanisms by means of biochemistry, pharmacological and behavioral testing, and neuronal functional imaging.

## 2. Materials and Methods

### 2.1. Animals

Sprague-Dawley rats (male and female, 3-4 months of age) were bought from Shanghai Laboratory Animal Center of China. All animals were housed in Zhejiang Chinese Medical University Laboratory Animal Center (5 animals/cage, 12 h dark-light cycle, 24 ± 2°C). Animals were given free access to water and food. All animals were allowed at least 1 week to adjust to the new breeding facility before any test. Animals were randomly allocated using random number tables. A total of 180 rats were used in this study. The group size in our experiments was chosen based upon our previous experience and studies using similar experimental protocols. All experimental procedures were carried out in accordance with the National Institutes of Health *Guide for the Care and Use of Laboratory Animals* (NIH Publications No. 8023, revised 1978), and the procedures were all approved by Animal Ethics Committee, Zhejiang Chinese Medical University (# ZSLL2017-183).

### 2.2. Animal Model of CRPS-I Establishment

Chronic postischemia pain model was set up via imposing ischemia and reperfusion to the hind limb of the rat [7, 8]. The rats were anesthetized by injecting 50 mg/kg (i.p.) sodium phenobarbital.

7/32 (5 mm) internal diameter O-ring was used to ligate the hind limb at a position near ankle joint for 3 h. Three hours later, the O-ring was removed. The control group of rats was treated with the same anesthesia steps but without O-ring ligature. No analgesic was provided during model establishment since it could feasibly interfere with the development of the pain state of this animal model.

### 2.3. Paw Edema Measurement

The edema of the hindpaw was measured by the digital caliper as previously reported [19]. The percent increase in paw thickness was used to indicate the paw edema. Three measurements were taken for each rat, and the average value was obtained thereafter.

### 2.4. Mechanical Allodynia

Mechanical allodynia was measured by methods described before [20]. Briefly, each rat was allowed to acclimate to testing conditions for 30 min beforehand. Mechanical allodynia was measured by applying *von* Frey filaments to the midplantar surface of the rat's hindpaw. The “Up and Down” method was used for measurement and calculation of 50% paw withdrawal threshold [21, 22]. The experimenter for behavioral test was blinded during the allocation, the conduct of the experiment, the outcome assessment, and the data analysis procedures.

### 2.5. Drug Treatment

N-Acetyl cysteine (NAC, Beyotime Biotechnology, China) was diluted in PBS and daily administered through intraperitoneal injection (200 mg/kg, i.p.) 30 min ahead of behavior test. The dosage of NAC is based upon previous literatures [23, 24]. H_2_O_2_ was diluted in PBS and injected (100 nmol/50 *μ*l) to dorsal region of hindpaw. The control group of rats was injected with vehicle (PBS) only. ML385 (Tocris, USA) was dissolved in DMSO and further diluted in PBS for local hindpaw injection (400 *μ*g/site in 50 *μ*l volume). ML385 was injected 30 min before each EA intervention.

### 2.6. EA Procedure

The same EA procedure was adopted in the present study from our recent reports, with some minor modifications [10]. Briefly, the animals were immobilized gently with a self-made retainer. Four acupuncture needles were inserted into bilateral ST36 and BL60 acupoints (with a depth of around 5 mm). The HANS-200A Acupoint Nerve Stimulator (Huawei Co., Ltd., China) was used to connect the acupuncture needles. 2/100 Hz alternative square wave with intensities from 0.5-1.5 mA was administered for 30 min per session. For animals receiving sham EA intervention, the acupuncture needle was only subcutaneously inserted (at a depth of 1 mm) into bilateral BL60 and ST36, and no electrical stimulation was applied. The EA or sham EA interventions were performed on animals on a daily basis for 7 days.

### 2.7. Tissue Collection and RNA Extraction

CPIP or control group rats were anesthetized deeply using sodium pentobarbital at dosage of 40 mg/kg. The animals were then perfused with 0.9% saline with 4°C. Then, the ipsilateral hindpaw skin was harvested and immediately preserved in RNAlater solution (Thermo Fisher, USA). TRIzol reagent (Thermo Fisher, USA) with DNase I was used for total RNA extraction. The concentrations and the purities of skin samples were evaluated using the NanoDrop Spectrophotometer (NanoDrop Products, USA). RNA integrity number (RIN) was determined by the Agilent TapeStation System (Agilent Technologies, USA).

### 2.8. RNA-Seq Library Establishment and RNA-Seq

Total mRNAs from 3 rats of the CPIP and control groups were isolated and used to construct sequencing libraries. The mRNA molecules were purified from total RNA using oligo (dT)-attached magnetic beads. The mRNAs were fragmented into small pieces using fragmentation reagent. First-strand cDNA was generated using random hexamer-primed reverse transcription and then was followed by a second-strand cDNA synthesis. The synthesized cDNA was subjected to end-repair and then was 3′ adenylated. Adaptors were ligated to the ends of these 3′ adenylated cDNA fragments. This process is to amplify the cDNA fragments with adaptors from the previous step. PCR products are purified with the SPRI beads and dissolved in EB solution. The double-stranded PCR products were heat denatured and circularized by the splint oligosequence. The single-strand circle DNA (ssCir DNA) was formatted as the final library. Library was validating on the Agilent Technologies 2100 bioanalyzer. The library was amplified with phi29 to make DNA nanoball (DNB) which has more than 300 copies of one molecular. The DNBs were loaded into the patterned nanoarray, and single-end 50 base reads were generated in the way of sequencing by synthesis. Finally, the fragments were enriched by PCR amplification to construct a library ready for sequencing using BGISEQ-500 by BGI (Shenzhen, China).

### 2.9. Bioinformatics Analysis

Primary sequencing data produced by RNA-Seq (raw reads) were subjected to quality control (QC). Raw data were filtered into clean reads by internal software SOAPnuke (version 1.5.2) as follows: remove reads in which unknown bases (N) are more than 10%; remove reads with adaptors; remove low-quality reads (we define the low-quality read as the percentage of base in which quality is lesser than 15 and greater than 50% in a read). QC of alignment was performed to determine if resequencing was needed. If the alignment result passed QC, downstream analysis includes gene expression, differentially expressed genes, and cluster analysis.

### 2.10. Cluster Analysis and Screening of Differentially Expressed Genes

Distances of expressed genes were calculated using the Euclidean method [25]. The sum of the squared deviation algorithm was used to calculate distance. The cluster analysis and heat map visualization of gene expression patterns were performed using the “pheatmap” package in the R software of Bioconductor. Differentially expressed mRNAs with statistical significance were identified through Scatter Plot filtering as we reported before [26]. The threshold required for the results to be considered significant was as follows: *q* value ≤ 0.05 and absolute value of ∣log_2_ (fold change) | ≥0.585.

### 2.11. Determination of Oxidant/Antioxidant Status

Ipsilateral hindpaw tissues from each group were collected. The samples were then homogenized, followed by centrifugation at 3,000 rpm for 15 min. Supernatant was collected for superoxide dismutase (SOD), reduced glutathione (GSH), malondialdehyde (MDA), and hydrogen peroxide (H_2_O_2_) assay as we described before [23, 27].

### 2.12. qPCR Testing and Analysis

RNA from the ipsilateral hindpaw skin samples was extracted by TRIzol reagent (Thermo Fisher, USA). 1000 ng of total RNA from hindpaw tissues was reversely transcribed with Takara PrimeScript™ Master Mix kit to generate cDNAs. qPCR reactions were performed using the FastStart Universal SYBR® Green Master PCR kit (Takara Bio Inc., China). Three replicates were performed for each sample. The gene expression level was normalized to *β*-actin, the housekeeping control gene. CFX96 Real-Time System software was used for CT value determination and analysis. Relative mRNA expression levels were determined by the well-established 2*^ΔΔ^*CT methodology. For detailed information regarding the sequences for the primers, please check our Suppl. Table 1.

### 2.13. Immunofluorescence Staining

Rats were anesthetized and perfused transcardially with 4°C saline followed by 4% fresh paraformaldehyde. The ipsilateral hindpaw skin and the ipsilateral spinal cord were harvested, fixed for 4 h in 4% paraformaldehyde, and gradient dehydrated in sucrose solution (30%) overnight at 4°C. Samples embedded in a frozen microtome (Thermo NX50, Thermo Fisher, USA) were cut into frozen sections with the thickness for 14 *μ*m of hindpaw skin and 25 *μ*m of the spinal cord. Then, samples were mounted onto gelatin-coated glass slides for immunofluorescence. The sample sections were blocked in normal donkey serum (5%) in Tris-buffered saline with Tween (TBST) for 1 h at room temperature and then incubated with corresponding primary antibodies overnight at 4°C. The primary antibody was applied to immunostain the hindpaw skin sample sections: mouse anti-8-OHG (1 : 800, ab62623, Abcam), and the primary antibodies were applied to immunostain spinal cord sample sections: rabbit anti-c-Fos (1 : 800, #2250, CST), mouse anti-GFAP (1 : 1000, #c9205, Sigma), and mouse anti-OX42 (1 : 1000, #ab1211, Abcam). These antibodies have successfully been used in previous literatures for immunofluorescence staining of corresponding targets [28–31]. Then, the sample sections were labeled with the fluorescent secondary antibodies (Cy3-, Cy5-, or FITC-conjugated) for 1 h at room temperature after washing in the dark. The pictures of sample sections were captured by a laser scanning confocal microscope (Nikon A1R, Japan). Images were captured with uniformed settings, and experimenters were blinded to treatment groups for image quantification. Three sections from each animal were selected, averaged, and then compared among groups as described previously in our studies [32, 33].

### 2.14. Western Blotting

The procedures are described in our previous study [27]. Briefly, the ipsilateral hindpaw tissues were collected quickly on ice after the rats being anesthetized with sodium pentobarbital (40 mg/kg, i.p.). The tissues were then homogenized in RIPA lysis buffer and centrifuged at 15,000 rpm for 15 min at 4°C. The supernatants were collected and measured the protein concentration using a BCA protein assay kit (Thermo Fisher, USA). Equal amounts of protein samples (10 *μ*g) were electrophoresed on 5%-12% SDS-polyacrylamide gels and transferred onto PVDF membranes (Bio-Rad, USA). After the membranes were blocked by 5% nonfat milk in TBST solution for 1 h at room temperature, the primary antibodies were applied overnight at 4°C and the HRP-coupled secondary antibodies were applied for 2 h at room temperature. The antibodies used in the present study were listed as follows: *β*-actin (1 : 5000, mouse monoclonal, #ab20272, Abcam), 4-hydroxynonenal (4-HNE, 1 : 800, rabbit polyclonal, #ab46545, Abcam), and nuclear factor erythroid 2-related factor 2 (Nrf2, 1 : 1000, #rabbit polyclonal, R-1312, HUABIO). 4-HNE and Nrf2 antibodies have been successfully used in previous studies for protein detection [23, 34]. The expression levels of targeted protein are normalized to the density of *β*-actin. The original full blot images can be found in Suppl. Fig. 1.

### 2.15. DRG Harvest and Ca^2+^ Imaging

Ca^2+^ imaging was performed as we described before [35]. Briefly, DRG were isolated from ipsilateral lumbar L4-L6 segment from each rat. Samples were not pooled from multiple rats, and a total of 15 rats were used in this study. The DRG were dissociated by collagenase (1 mg/ml) and dispase (2 mg/ml) (Gibco, Thermo Fisher) and incubated in DMEM +10% FBS and used 4-6 h after being harvested. Cells were first washed 3 times with the loading buffer which contains 140 mM NaCl, 5 mM KCl, 2 mM CaCl_2_, 2 mM MgCl_2_, and 10 mM HEPES (pH 7.4 adjusted with NaOH). Then, the cells were incubated with 10 *μ*M Fura 2-AM at 37°C for 30 min in the loading buffer. After the incubation, the cells were rinsed with loading buffer for 3 times and rest for another 30 min at 37°C in the dark. Then, the cells were subject to Ca^2+^ imaging procedure. Ca^2+^ imaging was undertaken by a Nikon ECLIPSE Ti-S microscope. Orca Flash 4.0 CCD camera (Hamamatsu, Japan) was used for capturing images. Polychrome V monochromator (Till Photonics, USA) was used as the light source to generate 340 and 380 nm ultraviolet light. The fluorescence images were further analyzed and processed by the ImageJ software. A criterion was set up to distinguish whether a cell is responsive in Ca^2+^ imaging test, namely, when the peak Ca^2+^ signal of the cell reaches over 20% of the baseline value, then the cell is considered as a positively responding cell [36, 37].

### 2.16. Regional Blood Flow Measurement by Laser Doppler

The rats were anesthetized with isoflurane before laser Doppler measurement. Anesthesia is first achieved with 3–4% isoflurane supplied with oxygen, and then, the anesthesia was further maintained with constant 1–2% isoflurane supplied with oxygen. The rats were laid on a thermal pad maintained constantly at 37°C. The PeriFlux System 5000 Laser Doppler Flow Meter (Perimed, Sweden) was used to measure regional blood flow in the affected hindpaws of rats. The whole area of the hindpaw was defined as the region of interest, and the blood flow was then calculated using the software provided by PeriFlux System (Perimed, Sweden).

### 2.17. Statistical Analysis

SPSS 19.0 (IBM Corp., USA) was used for all statistical analysis through the study. All data were included in data analysis and presentation. Data was presented as the mean ± SEM. The complete statistical results (including mean, SEM, SD, and confidence interval) are presented in Suppl. Table 2. To compare data between 2 groups, Student's *t*-test was applied for statistical analysis. To compare data among 3 or more groups, one-way or two-way analysis of variance (ANOVA) with Tukey's post hoc test was applied for statistical analysis. In cases when the data was nonnormally distributed (tested by Kolmogorov-Smirnov test), a nonparametric test (e.g., Mann-Whitney test) was used for analysis. Statistical significance was accepted at a level of *p* < 0.05.

## 3. Results

### 3.1. RNA-Seq Revealed Oxidation-Reduction Process Is Markedly Affected in Hindpaw Tissues of a Rat Model of CRPS-I

**Figure 1:**
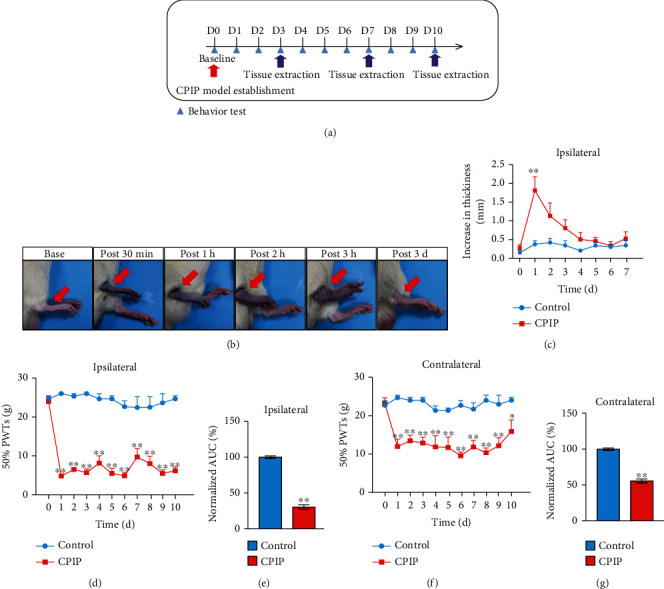
The rat CPIP model showed persistent bilateral mechanical allodynia in the hindpaw. (a) Experimental protocol for establishing the rat chronic postischemic pain (CPIP) model. (b) Representative pictures showing the rat's hindpaw during and after CPIP model establishment. The red arrow indicates the paw with the O-ring treatment (ipsilateral side). (c) Paw thickness evaluation of control and CPIP model rats of ipsilateral side. (d) 50% paw withdrawal threshold (PWT) measurement of ipsilateral hindpaw. (e) Summary of the normalized area under the curve (AUC) as in (d). (f) 50% PWT measurement of contralateral hindpaw. (g) Summary of the normalized area under the curve (AUC) as in (f). *n* = 6 rats/group. ^∗∗^*p* < 0.01 and ^∗^*p* < 0.05 vs. the control group.

**Figure 2:**
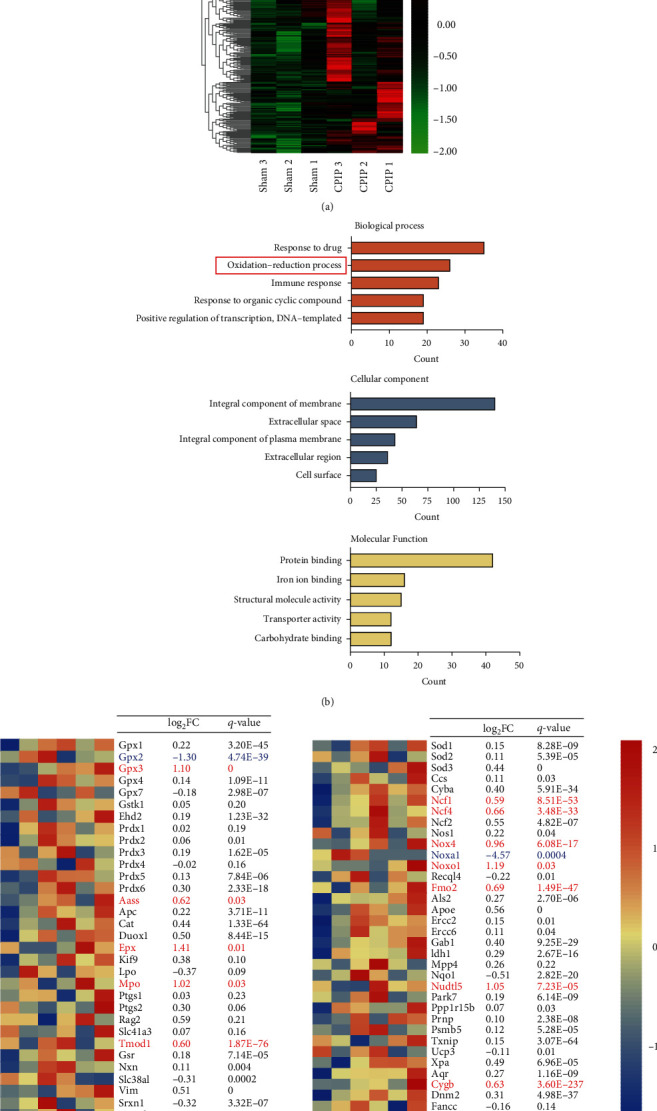
RNA-Seq profiling of gene expression in local ipsilateral hindpaw tissues of CPIP model rats. (a) Heat map showing hierarchical clustering patterns of DEGs from the control and CPIP model groups. (b) Gene Ontology (GO) pathway analysis of DEGs. The top 5 mostly enriched pathways were illustrated. (c) Heat map summarizing the genes particularly involved in oxidative stress, antioxidant defense, and reactive oxygen metabolism process as well as corresponding log_2_FC (FC: fold change) and *q* value. Upregulated DEGs are displayed in red, whereas downregulated are in blue. Non-DEGs are in black. *n* = 3 rats/group.

The rat CPIP model that mimics human CRPS-I was established according to the methods before [7, 8]. The ligated hindpaws of the model rats displayed obvious signs of tissue cyanosis and edema as shown in Figures 1(a) and 1(b) during ligation. The edema lasted over two days and returned back to normal (Figure 1(c)). Remarkable bilateral mechanical pain allodynia was observed in model rats compared with control rats that lasted until the endpoint of our study (>10 days) (Figures 1(d)–1(g)). We aimed to explore potential genes or biological process involved in mediating the pain response of CPIP model rats. Therefore, we performed gene expression profiling by RNA-Seq to explore potential gene expression changes in ipsilateral hindpaw tissues from CPIP rats vs. control rats. The ipsilateral hindpaw tissues were collected 7 d after CPIP model establishment. We successfully extracted high-quality RNA from hindpaw tissues for RNA-Seq analysis (Suppl. Fig 2). A total of 226 (upregulated) and 192 genes (downregulated), amounting to 1.48% and 1.26% of all genes being sequenced (15,251), respectively, were identified to be differentially expressed genes (DEGs) in hindpaw tissues from CPIP rats vs. control rats. We overlaid these DEGs in a heat map and performed cluster analysis to further examine data consistency between and within groups (Figure 2(a)). The cluster analysis indicated that the dataset within the CPIP or control groups exhibited high consistency, whereas the two groups are clearly separated (Figure 2(a)). We next performed Gene Ontology (GO) analysis of the DEGs we have identified.  Figure 2(b) shows the top 5 most enriched pathways in each GO section. Of particular interest was “oxidation-reduction process,” which ranked the 2^nd^ among all biological process. Oxidative stress is a factor that is considered to be involved triggering chronic pain [38]. We further chose some representative genes known to contribute to oxidative stress, antioxidant defense, and reactive oxygen metabolism [39] and evaluated the impact of CPIP model on these genes. Among the 71 genes tested, 14 were found to be significantly affected (Figure 2(b) and Suppl. Table 3), demonstrating oxidative stress may exist in local hindpaw tissues of CPIP rats.

### 3.2. Oxidative Stress Occurred Primarily in Local Hindpaw Tissues of CPIP Rats and Contributes to Mechanical Allodynia

**Figure 3:**
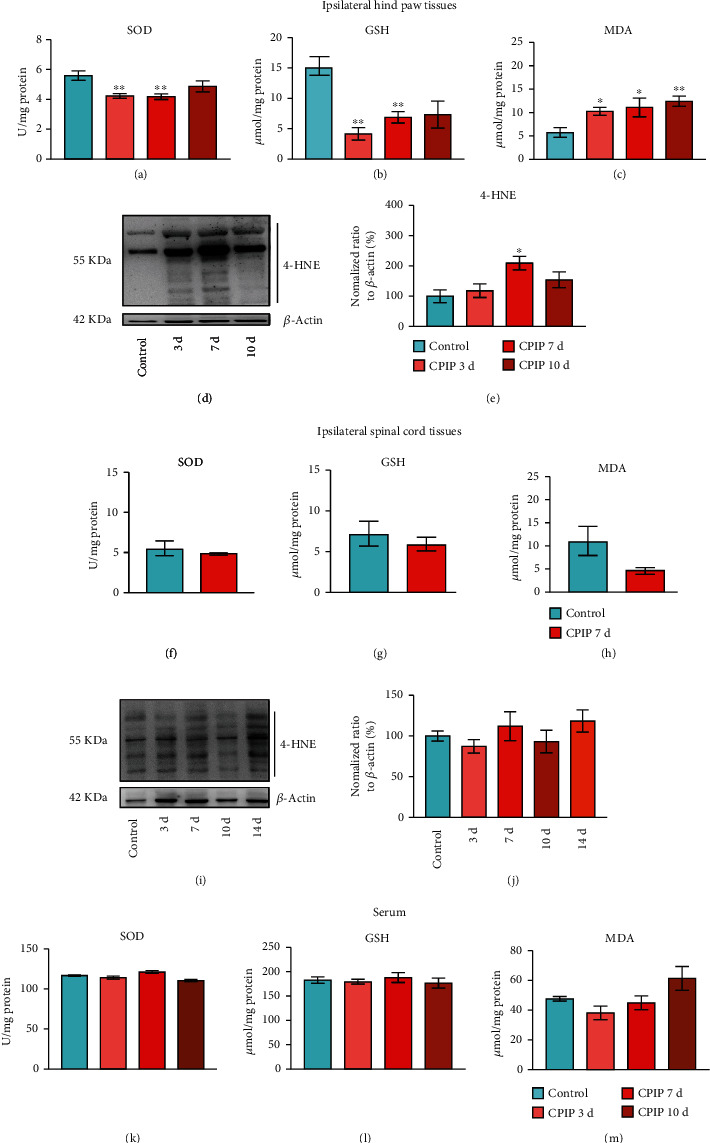
Evaluating oxidative stress status in ipsilateral hindpaw, the spinal cord, and serum of CPIP rats. (a–c) Results showing SOD activity (a), GSH-Px activity (b), and MDA content (c) determined in ipsilateral hindpaw tissues from control and CPIP model rats 3, 7, and 10 d after model establishment. (d) 4-HNE expression in ipsilateral hindpaw tissues determined by Western blotting at time points as indicated. Upper panel shows representative images of 4-HNE and lower panel shows *β*-actin. (e) Summarized results of 4-HNE expression normalized to *β*-actin. Results showing SOD activity (f), GSH-Px activity (g), and MDA content (h) determined in ipsilateral spinal cord tissues from control and CPIP model rats 7 d after model establishment. (i) 4-HNE expression in ipsilateral spinal cord tissues determined by Western blotting at time points as indicated. The upper panel shows representative images of 4-HNE, and the lower panel shows *β*-actin. (j) Summarized results of 4-HNE expression normalized to *β*-actin. Results showing SOD activity (k), GSH-Px activity (l), and MDA content (m) determined in serum from control and CPIP model rats 3, 7, and 10 d after model establishment. *n* = 5‐6 rats/group.

We continued to study the oxidative stress in hindpaw tissues of male CPIP rats in more detail. We evaluated the changes of SOD and GSH-Px enzyme activities, two antioxidant enzymes. We also checked the contents of 4-hydroxynonenal (4-HNE) and malondialdehyde (MDA), two lipid peroxidation products in ipsilateral hindpaw tissues. As shown in Figures 3(a) and 3(b), we found that SOD and GSH levels were significantly decreased at days 3 and 7, whereas the content of MDA was significantly increased through day 10 (Figure 3(c)). As shown in Figures 3(d) and 3(e), the content of 4-HNE was significantly increased at day 7 among CPIP rats. We proceeded to evaluate the oxidative stress status in spinal cord tissue and serum of CPIP model rats. SOD, GSH, and MDA levels were not significantly changed at day 7, and 4-HNE level remained unchanged through day 14 in the ipsilateral spinal cord of CPIP model rats (Figures 3(f)–3(j)). In addition, the immunoactivity of 8-OHG, a marker of oxidative damage in cellular nucleic acids, remains unchanged in the spinal cord during observation (till day 10, Suppl. Fig. 3A-C). Similarly, SOD, GSH, and MDA levels remained unchanged through day 10 in CPIP rat serum (Figures 3(k)–3(m)). Therefore, these data suggest that the oxidative stress occurred primarily in local hindpaw tissues of CPIP rats.

**Figure 4:**
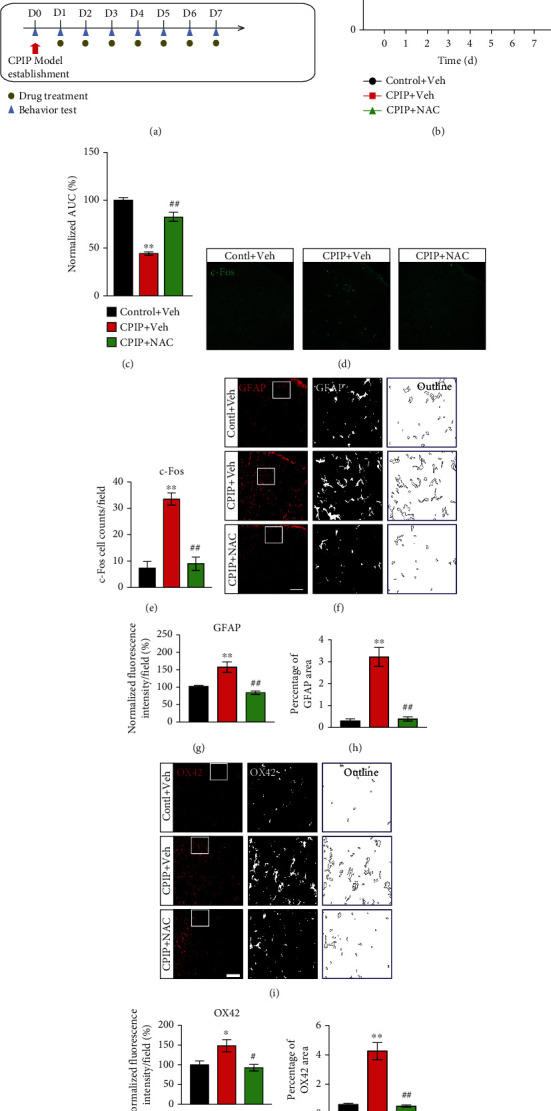
NAC treatment ameliorates mechanical allodynia of CPIP rats. (a) Schematic protocol showing time points for model establishment and NAC/vehicle treatment. (b) Time course of 50% PWT changes after NAC/vehicle treatment. The CPIP+NAC group receives daily NAC (200 mg/kg, i.p.) treatment, whereas the CPIP+Veh group receives vehicle (PBS, i.p.) treatment. (c) Summary of AUC as in (b). (d) Ipsilateral SCDH with c-Fos staining from the control+Veh, CPIP+Veh, and CPIP+NAC groups. (e) Summary of the number of c-Fos-positive cells per observation field. (f) Ipsilateral SCDH with GFAP staining. Summary of the normalized fluorescence intensity (%) of GFAP (g) and percentage of GFAP-stained area (h). (i) Ipsilateral SCDH with OX42 staining. Summary of the normalized fluorescence intensity (%) of OX42 (j) and percentage of OX42-stained area (k). Scale bar = 100 *μ*m. *n* = 5‐6 rats/group. ^∗^*p* < 0.05 and ^∗∗^*p* < 0.01 vs. the control+Veh group. ^#^*p* < 0.05 and ^##^*p* < 0.01 vs. the CPIP+Veh group.

We then tested if the oxidative stress contributes to pain mechanisms of the rat model of CRPS-I. CPIP rats were administered with antioxidant NAC on a daily basis (200 mg/kg, i.p., Figure 4(a)). NAC administration effectively reduced the mechanical allodynia of ipsilateral hindpaw (Figure 4(b)). AUC analysis demonstrated an accumulated alleviation of mechanical allodynia in the CPIP+NAC group vs. CPIP+Veh group (Figure 4(c)). We then checked the effects of NAC treatment on spinal glial and neuron activation, which are critical processes contributing to central pain sensitization during chronic pain [40, 41]. Immunostaining experiments unraveled that c-Fos-labeled cell number was markedly increased in ipsilateral spinal cord dorsal horn (SCDH) of the CPIP+Veh group vs. control+Veh group (Figures 4(d) and 4(e)). In addition, the immunostaining intensity of two markers for microglia and astrocyte, namely, OX42 and GFAP, was markedly increased in ipsilateral dorsal horn of the CPIP+Veh group vs. control+Veh group as well (Figures 4(f)–4(k)). More importantly, the magnitudes of these cellular events were all reduced with NAC treatment (Figures 4(d)–4(k)).

**Figure 5:**
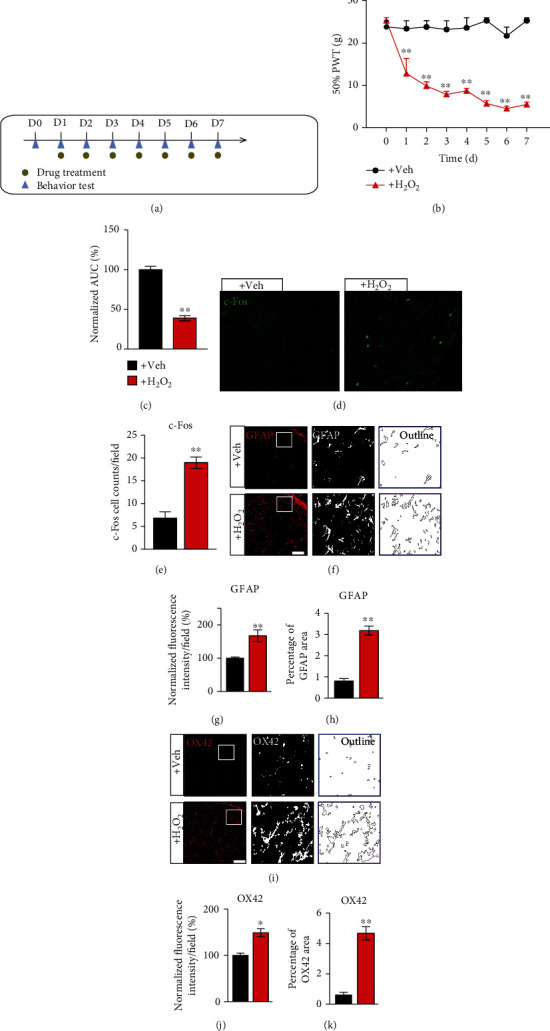
Locally increasing oxidative stress produces mechanical allodynia among naïve rats. (a) Schematic protocol showing time points for H_2_O_2_/vehicle treatment in naïve rats. (b) Time course of 50% PWT changes after H_2_O_2_/vehicle treatment. The +H_2_O_2_ group receives daily H_2_O_2_ (100 nmol/site) injection into hindpaws, whereas the +Veh group receives only vehicle (PBS) injection. (c) Summary of AUC as in (b). (d) Ipsilateral SCDH with c-Fos staining from the +Veh vs. +H_2_O_2_ group. (e) Summary of the number of c-Fos-positive cells per observation field. (f) Ipsilateral SCDH with GFAP staining. Summary of the normalized fluorescence intensity (%) of GFAP (g) and percentage of GFAP-stained area (h). (i) Ipsilateral SCDH with OX42 staining. Summary of the normalized fluorescence intensity of OX42 (j) and percentage of OX42-stained area (k). *n* = 6 rats/group. ^∗∗^*p* < 0.01 vs. +Veh group.

We then tested whether locally increasing oxidative stress could mimic the chronic pain response of CPIP model rats. Daily local injection of H_2_O_2_ (an endogenous ROS product, 100 nmol/site), into the hindpaws of naïve rats, elicited persistent mechanical allodynia (Figures 5(a) and 5(b)). AUC analysis indicated an accumulated development of mechanical allodynia in animals treated with H_2_O_2_ vs. vehicle (Figure 5(c)). In addition, immunostaining experiments indicated that persistent H_2_O_2_ injection produced c-Fos activation (Figures 5(d) and 5(e)), as well as GFAP and OX42 overactivation (Figures 5(f)–5(k)) in ipsilateral SCDH of treated rats. This suggests that persistent local oxidative stress per se can cause mechanical allodynia, spinal neuron, and glial cell activation, which mimics chronic pain and spinal cellular events of CPIP model rats.

Sex-dependent pain mechanisms have been reported in certain pain conditions. We then evaluated the oxidative status in female CPIP model rats. As shown in Suppl. Fig. 4A-D, we found that SOD and GSH levels were significantly decreased, whereas H_2_O_2_ and MDA level was significantly increased in ipsilateral hindpaw tissues at day 7 in female CPIP model rats. By contrast, the contents of these substances were not obviously changed from the ipsilateral spinal cord (Suppl. Fig. 4E-H). Similar to male rats, NAC effectively ameliorated the mechanical hypersensitivity of ipsilateral hindpaws from female rats (Suppl. Fig. 4I). This result suggests that local oxidative stress can drive pain response in CPIP model animals in both sexes. In all, the above results demonstrate a critical role of local oxidative stress in hindpaw tissue that contributes to chronic pain mechanisms of a rat model of CRPS-I.

### 3.3. Electroacupuncture Alleviates Mechanical Allodynia of CPIP Rats through Reducing Local Oxidative Stress and Inflammation in Hindpaw Tissues

**Figure 6:**
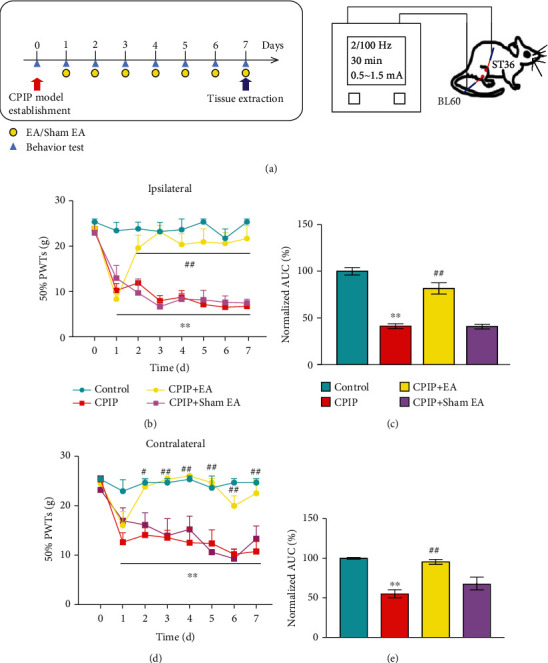
EA intervention ameliorates mechanical allodynia of CPIP rats. (a) Schematic protocol illustrating time points for EA or sham EA treatment in CPIP model rats (left) and schematic picture showing EA parameters and the locations of ST36 and BL60 acupoints on the rat (right). (b) Time course of the effect of EA/sham EA treatment on 50% PWT of ipsilateral hindpaws of CPIP model rats. (c) Summary of AUC as in (b). (d) Time course of the effect of EA/sham EA treatment on 50% PWT of contralateral hindpaws. (e) Summary of AUC as in (d). *n* = 6 rats/group. ^∗∗^*p* < 0.01 vs. the control group. ^##^*p* < 0.01 vs. the CPIP+sham EA group.

**Figure 7:**
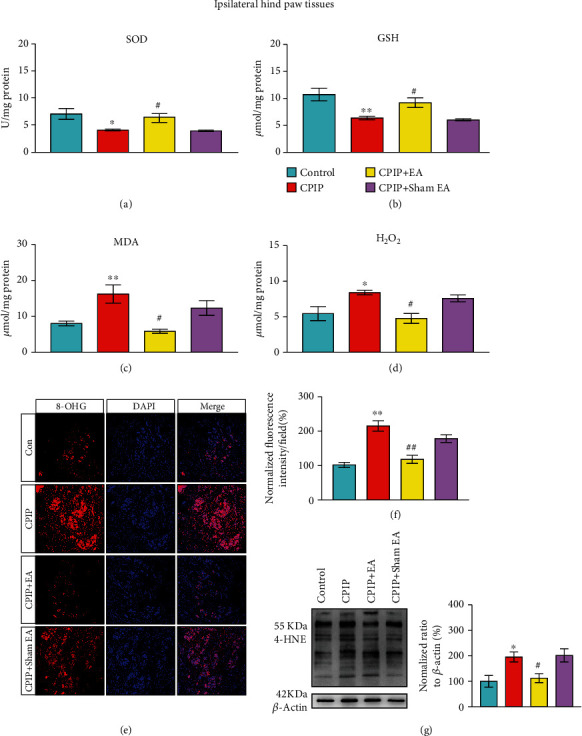
EA intervention ameliorates excessive oxidative stress in local hindpaw tissues of CPIP rats. Results showing SOD activity (a), GSH-Px activity (b), MDA content (c), and H_2_O_2_ content (d) determined in ipsilateral hindpaw tissues of the control, CPIP, CPIP+EA, and CPIP+sham EA groups 7 d after model establishment. (e) Representative pictures showing 8-OHG immunostaining in ipsilateral hindpaw tissues of each group. (f) Summarized data of normalized fluorescence intensity of 8-OHG per observation field of each group. (g) 4-HNE expression determined by Western blot in ipsilateral hindpaw tissues of each group. *n* = 6 rats/group. ^∗^*p* < 0.05 and ^∗∗^*p* < 0.01 vs. the control group. ^#^*p* < 0.05 and ^##^*p* < 0.01 vs. the CPIP+sham EA group.

Electroacupuncture (EA), a modified acupuncture therapy, is effective for pain management without obvious adverse effects [42]. EA showed positive effects on alleviating pain, improving limb dysfunction, and improving daily activities of CRPS-I patients [18]. Our recent study further demonstrated EA effectively alleviated mechanical allodynia of CPIP model rats and identified 2/100 Hz as an optimal therapeutic frequency [10]. However, the analgesic effects of EA on CRPS-I still remain elusive. Therefore, we employed 2/100 Hz EA on ipsilateral acupoints of BL60 and ST36 from CPIP model rats as we did in our recent study (Figure 6(a)) [10]. We found that daily EA treatment (for successive 7 days) effectively ameliorated bilateral mechanical pain hypersensitivities of CPIP rats (Figures 6(b)–6(e)), whereas sham EA had no effect, a result consistent with our recent study [10]. We proceeded to examine if the antiallodynic effect exerted by EA was possibly attributed to the modulation of local oxidative stress in the hindpaw. Figure 6(a) shows the experimental scheme and the time points for observation and tissue collection. Oxidative stress evaluation indicated that SOD and GSH levels were decreased significantly, whereas MDA and H_2_O_2_ levels were increased significantly in ipsilateral hindpaw tissues from model rats vs. control rats (Figures 7(a)–7(d)). EA intervention significantly increased the SOD and GSH levels, while it decreased MDA and H_2_O_2_ overproductions vs. the sham EA group (Figures 7(a)–7(d)). 8-OHG immunoactivity was significantly increased in local hindpaw tissues from CPIP rats, and the intervention of EA significantly alleviated its overexpression (Figures 7(e) and 7(f)). Immunoblot experiments showed that 4-HNE overproduction from hindpaw tissues of CPIP rats was significantly attenuated by EA intervention, whereas sham EA had no such effect (Figure 7(g)).

Our study suggests that persistent local oxidative stress triggers the activation of glial cells in SCDH. We then continued to investigate if EA might attenuate these cellular events in the spinal cord, which constitutes critical process leading to central pain sensitization [43]. GFAP and OX42 immunoreactivity in SCDH rose markedly from model group vs. controls (Suppl. Fig. 5A-F). Electroacupuncture effectively reduced GFAP and OX42 immunoreactivity increase in ipsilateral SCDH (Suppl. Fig. 5A-F).

**Figure 8:**
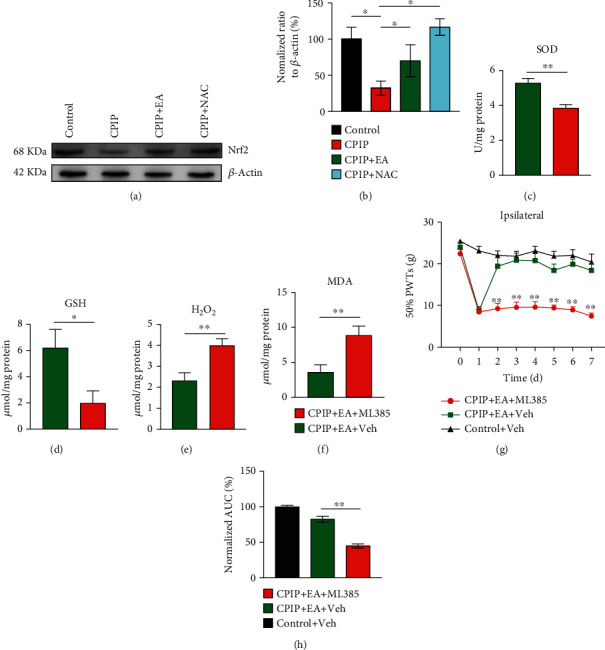
EA exerts antiallodynic effect on CPIP rats through activating Nrf2 in affected hindpaw. (a) Nrf2 expression determined by Western blotting in ipsilateral hindpaw tissues of the control, CPIP, CPIP+EA, and CPIP+NAC groups 7 d after model establishment. The upper panel shows representative images of Nrf2, and the lower panel shows *β*-actin. (b) Summarized results of Nrf2 expression normalized to *β*-actin. Evaluation of SOD activity (c), GSH-Px activity (d), H_2_O_2_ (e), and MDA content (f) in ipsilateral hindpaw tissues from the CPIP+EA+ML385 and CPIP+EA+Veh group rats 7 d after model establishment. (g) Time course of the effect of intraplantar injecting Nrf2 antagonist ML385 (400 *μ*g/rat) or corresponding vehicle (1% DMSO in PBS) on 50% PWT of ipsilateral hindpaws of CPIP model rats. (h) Summary of AUC as in (g). *n* = 6 rats/group. ^∗^*p* < 0.05 and ^∗∗^*p* < 0.01 vs. CPIP+EA+Veh or as indicated.

We then investigated how EA may modulate local oxidative stress in CPIP model rats. Nrf2 is a nuclear transcription factor that couples with antioxidant response element and coordinates with the activation of antioxidative genes and pathways [44]. Next, to investigate if EA may produce antioxidative effects through Nrf2 activation, we first examined Nrf2 expression. As shown in Figures 8(a) and 8(b), the protein level of Nrf2 was markedly reduced in hindpaw tissues of CPIP rats vs. the control group, whereas EA treatment restored Nrf2 expression. Antioxidant NAC produced similar effects on Nrf2 as EA (Figures 8(a) and 8(b)). Local application of Nrf2-specific antagonist ML385 abolished the antioxidative effects of EA by reducing GSH and SOD levels and increasing H_2_O_2_ and MDA levels in hindpaw tissues (Figures 8(c)–8(f)). Furthermore, ML385 treatment effectively abolished EA-mediated antiallodynia in CPIP rats (Figures 8(g) and 8(h)). Taken together, these findings indicate that EA alleviates mechanical pain response of CPIP rats by reducing local oxidative stress via Nrf2-dependent antioxidative mechanism.

### 3.4. EA Reduced the Upregulation of Inflammatory Cytokines in Hindpaw Tissues and Reduced the Enhanced TRPA1 Channel Activity in Peripheral Sensory Neurons Innervating Hindpaw of CPIP Rat

Oxidative stress triggers the activation of a variety of transcription factors, resulting in upregulation of an array of inflammatory mediators [45]. We found that the gene expressions of some typical inflammatory cytokines, *Tnfa*, *Il1b*, and *Il6*, were significantly upregulated in ipsilateral hindpaw tissues of CPIP model rats. The expressions of these inflammatory genes were all reduced by NAC (Suppl. Fig. 6A-C), indicating an important role of oxidative stress in mediating the upregulation of these inflammatory genes. In addition, EA treatment produced similar reduction of *Tnfa* and *Il1b* overexpression as NAC (Suppl. Fig. 6A&B).

**Figure 9:**
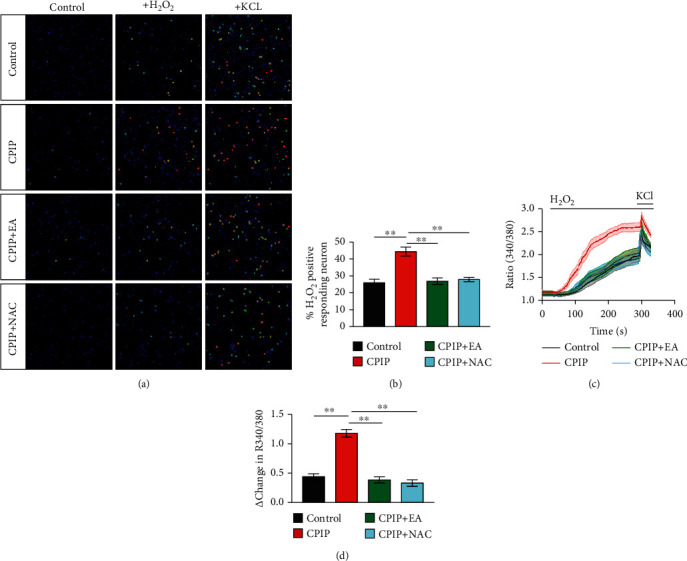
TRPA1 channel functional activity is enhanced in DRG neurons of CPIP rats, whereas repetitive EA or NAC treatment reversed TRPA1's enhanced activity. (a) Pseudocolor images from Fura-2-based ratiometric Ca^2+^ imaging. The pictures indicate Ca^2+^ responses from ipsilateral L4-6 DRG neurons derived from the control, CPIP, CPIP+EA, and CPIP+NAC group rats. H_2_O_2_ (500 *μ*M), an endogenous TRPA1 agonist, was used to stimulate TRPA1 in these neurons. KCl (40 mM) was applied to the bath at the end of each experiment to identify all live neurons. (b) Summary of the percentage of H_2_O_2_ positively responding neurons in each observation field from all groups as mentioned above. *n* = 6‐7 tests/group. Each group contains 150–200 neurons derived from 3–4 rats. (c) Comparison of averaged Ca^2+^ responses triggered by H_2_O_2_ among all groups. *n* > 40 cells/group. (d) Data summary showing Δ increase of peak 340/380 ratio. *n* > 60 neurons/group derived from 3-4 rats/group. ^∗∗^*p* < 0.01.

Many endogenous ROS molecules and lipid peroxidation products can target against neuronal TRPA1 channel in peripheral sensory neurons to produce nociception. IL-1*β* and TNF-*α* may contribute to pain sensitization via promoting TRPA1 channel expression [46, 47]. We then set to determine and compare the functions of TRPA1 channel from peripheral dorsal root ganglion (DRG) neurons under CPIP and control conditions. Ipsilateral L4-6 DRG neurons innervating the hind limbs were acutely dissociated from CPIP model and control rats. The cells were then incubated with Fura-2AM and subject to imaging of intracellular [Ca^2+^]. We found that CPIP treatment significantly increased the proportion of H_2_O_2_-responsive neurons among all neurons, as well as the magnitude of Ca^2+^ responses to H_2_O_2_ (Figures 9(a)–9(d)). EA intervention largely decreased the proportion of H_2_O_2_-responsive neurons as well as the magnitude of Ca^2+^ responses compared with sham EA (Figures 9(a)–9(d)). Meanwhile, NAC treatment produced similar effects as EA (Figures 9(a)–9(d)). These results suggest that the function of neuronal TRPA1 was enhanced under CPIP condition in a ROS-dependent manner, whereas EA intervention reverses the enhanced TRPA1 channel activities.

### 3.5. EA Improved Regional Blood Flow Dysfunction in Affected Hindpaws of CPIP Model Rats

**Figure 10:**
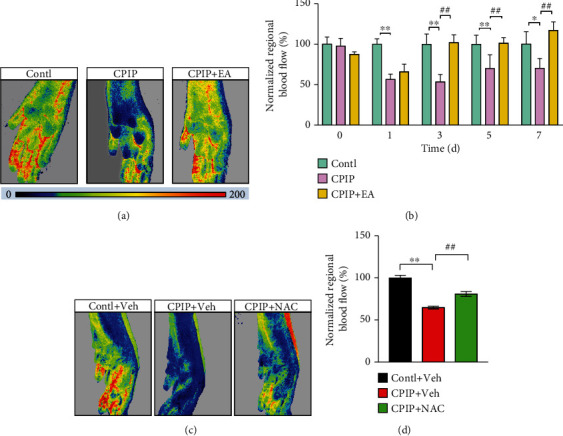
EA or NAC treatment improved regional blood flow dysfunction in affected hindpaws of CPIP model rats. (a) Representative pseudocolor images showing blood flow status in ipsilateral hindpaws of the control, CPIP, and CPIP+EA groups. (b) Time course summary of normalized blood flow of each group. Control group results were taken as 100%, and results of all other groups were normalized accordingly. (c) Representative pseudocolor images showing blood flow status in ipsilateral hindpaw tissues of the control+Veh, CPIP+Veh, and CPIP+NAC groups. (d) Summary of normalized blood flow of each group at day 7. ^∗^*p* < 0.05, ^∗∗^*p* < 0.01, and ^##^*p* < 0.01. *n* = 5‐7 rats/group.

CRPS-I patients showed microvascular injury and decreased blood flow in affected limbs [48]. These symptoms are among the key clinical features of CRPS-I patients. We then monitored the regional blood flow from the affected hindpaws of CPIP rats using laser Doppler. Results showed that CPIP model rats exhibited significantly decreased regional blood flow in hindpaw 1 d after model establishment and persisted until day 7 (Figures 10(a) and 10(b)). EA treatment significantly improved regional blood flow in hindpaw (Figures 10(a) and 10(b)). Moreover, NAC treatment produced similar improvement in blood flow as EA (Figures 10(c) and 10(d)). This result implies that regional blood flow dysfunction of CPIP model rats is ROS dependent and EA improves the dysregulated regional blood flow.

## 4. Discussion

Tissue inflammation and oxidative injury are considered important contributing factors to CRPS-I pathogenesis [49]. Antioxidants have shown pain-relieving effects on animal models of CRPS-I [7, 50, 51]. However, studies that systematically evaluated oxidative stress status in CRPS-I are still lacking. It remains unknown where the oxidative stress is predominantly generated and affected. In this study, we systematically evaluated oxidative stress status in a rat CRPS-I model, from the periphery to the spinal cord level. We found that oxidative stress occurred primarily in local affected hindpaw tissues but not in the spinal cord or serum of CPIP rats. These observations are relevant to one study showing oxidative stress occurred in hindpaw tissues of CPIP rats [13]. Moreover, oxidative stress in plasma of CRPS patients is not elevated compared to matched healthy volunteers [52]. We further found that locally increasing oxidative stress by applying H_2_O_2_ is sufficient to induce chronic pain and spinal glial cell overactivation in naive rats. These results suggest that ROS production in local affected hindpaw is an important mechanism contributing to the mechanical hypersensitivities among CPIP rats. These findings imply that targeting local oxidative stress in affected extremities might be effective approaches for relieving CRPS-I.

Emerging evidence indicates sex-dimorphic mechanism exists in driving certain chronic pain [53, 54]. Sex dimorphism in pain perception has gained increasing attention in both clinical and experimental studies. Epidemiology studies indicated females are more prone to develop CRPS-I than males [55]. Therefore, we tested whether local oxidative stress-induced pain in CPIP model animals was driven in a sex-dimorphic manner. We found that female CPIP model rats exhibit similar magnitude of oxidative stress increase in local affected hindpaw as male rats. No upregulation of oxidative stress in the spinal cord was observed in female rats. Moreover, NAC treatment attenuated pain response of female model rats in similar scale as male model rats. Therefore, these data suggest that local oxidative stress can drive pain response in CPIP model animals regardless of sex differences.

TRPA1 channel is distributed in a subgroup of peptidergic nociceptive neurons and can trigger neurogenic inflammation as well as nociception upon its activation. TRPA1 is known to be activated by endogenous ROS as well as lipid peroxidation products, including H_2_O_2_, 4-HNE, and OxPAPC [32, 56, 57]. 4-HNE has been identified to be increased in hindpaw tissues of CPIP model rats previously [13]. Our study further found that, in addition to 4-HNE, another ROS product H_2_O_2_ was increased in affected hindpaw tissues of CPIP model rats as well. Pharmacological blockage of TRPA1 has been shown to be effective for ameliorating pain in CPIP model animals [13]. Therefore, these two endogenous agonists of TRPA1 may work together to activate TRPA1 *in vivo* and contribute to pain mechanisms of CRPS-I. We further found that the functional TRPA1 channel activity was markedly upregulated in DRG neurons from CPIP rats. In addition, NAC or EA treatment significantly reduced the functional enhancement of TRPA1 channel. Oxidative stress triggers the activation of a variety of transcription factors, resulting in the upregulation of an array of inflammatory mediators. Specific inflammatory mediators, e.g., IL-1*β*, TNF-*α*, and IL-6, are capable of enhancing TRPA1 channel expression [46, 47]. The expressions of these cytokines were all increased in affected hindpaw tissues of CPIP model rats, but reduced by NAC or EA treatment. Thus, the enhanced functional TRPA1 channel activity in CPIP model rats may be attributed to ROS-dependent inflammatory cytokine release.

There are emerging evidences demonstrating the role of oxidative stress in chronic pain. ROS and lipid peroxidation products as a result of oxidative stress contribute to chronic pain via multiple mechanisms, including direct activation of nociceptor TRPA1 in the periphery and activation of microglia in the spinal cord, affecting spinal synaptic plasticity or producing pain-inducing substances [32, 58–60]. ROS scavengers or NADPH oxidase inhibitors, which reduce oxidative stress and ROS production, ameliorate chronic pain [61–64]. The Nrf2 antioxidant signaling is an endogenous machinery to counteract oxidative stress. Recently, several studies demonstrated that activating the Nrf2 antioxidant signaling exerts potent analgesic effects on chronic pain through alleviating ROS-associated pathological processes (e.g., inflammation and glial overactivation) and improving mitochondrial bioenergetics in the peripheral and/or central nervous system [65, 66]. Thus, activating the Nrf2 antioxidant signaling is a promising antinociceptive approach [67].

EA is an effective therapy to relieve pain and improve life quality of CRPS-I patients [18]. Our recent study demonstrated EA effectively reduces mechanical pain hypersensitivities in CPIP rats [10]. Here, we continued to explore how EA exerts therapeutic effects on CRPS-I. We found that EA significantly alleviated local oxidative stress in local hindpaw of CPIP rats and promoted antioxidant transcription factor Nrf2 upregulation. It has been reported that EA can restore the decreased levels of SOD and enhance Nrf2 expression in DRG of paclitaxel-induced peripheral neuropathic pain model rats [68]. However, it remains unknown whether EA-induced upregulation of Nrf2 contributes to its antioxidative and analgesic effect. In this study, we addressed this important question by testing the effects of pharmacological blocking Nrf2. We found that pharmacological blocking Nrf2 attenuated EA-induced antioxidative effects as well as analgesic effects. Therefore, our study established a causal relationship between EA-induced Nrf2 upregulation and EA-induced analgesic effect and proposed that Nrf2-mediated antioxidative effect contributes to EA's antiallodynic effect.

CPIP rats displayed contralateral mechanical allodynia, a sign of mirror image pain (MIP). At present, the pathogenesis of MIP remains largely unknown. Both central and peripheral mechanisms have been proposed to contribute to MIP [69]. It is found that glial cell showed overactivation in contralateral SCDH of CPIP rats, which could contribute to MIP [11, 14]. In addition, we recently found that contralateral DRG of CPIP rats showed multiple pain-related gene expression changes, indicating peripheral mechanisms may also contribute to MIP [70]. But how contralateral spinal glial cells and DRG are activated or affected during CPIP remains unknown. It is possible that the ipsilateral DRG or the spinal cord releases certain inflammatory mediators upon stimulation that can diffuse to the contralateral side via cerebrospinal fluid. Then, these inflammatory mediators may act upon contralateral spinal glial cells and DRG to produce MIP [71]. Since we also found that EA can ameliorate MIP of CPIP rats, thus, it will be tempting to explore whether EA could affect the abovementioned MIP mechanisms, both peripherally and centrally.

Tissue ischemia is a possible contributing factor for CRPS-I pathogenesis [72]. Ischemic tissue injury results in the production of ROS and inflammatory cytokines that can cause vascular disturbance and blood flow dysfunction [49]. Microvascular injury and blood flow dysfunction lead to tissue hypoxia and even more ROS production, all of which may contribute to pain [49, 73]. Impaired microcirculation and regional blood flow dysfunction in distal part of the affected extremity are important clinical features of CRPS-I [74, 75]. Here, we observed CPIP rats exhibit obvious regional blood flow dysfunction in affected hind limbs by laser Doppler, mimicking clinical feature of CRPS-I patients. NAC treatment improved the blood flow dysfunction of CPIP model rats, demonstrating an important contribution of ROS to its pathogenesis. Moreover, we found that EA treatment improved the regional blood flow dysfunction of CPIP model rats in similar degree as NAC. Since EA can produce similar antioxidative effect as NAC, we thus propose that the improvement of blood flow dysfunction by EA could be related with its antioxidative effect.

## 5. Conclusions

In all, we performed a systemic investigation of the oxidative stress status in a rat model for CRPS-I. We further identified local oxidative stress in affected hindpaw tissues as an important factor contributing to pathogenesis of CPIP model animals. EA can target against local oxidative stress by enhancing endogenous Nrf2-mediated antioxidative mechanism to relieve pain as well as inflammation in CRPS-I model animals. This work suggests targeting Nrf2 may be an effective way for relieving CRPS-I and EA is a promising method for CRPS-I management in clinical practice.

## Data Availability

The key data are contained in the figures, tables, and additional files. The datasets used and/or analyzed during this study can be further obtained from the corresponding authors on reasonable request.

## Supplementary Materials

Supplementary Materials The supplementary materials contain the following figures and tables in one file: Suppl. Figure 1: original Western blot images. Suppl. Figure 2: high-quality hindpaw tissue RNA obtained for RNA-Seq. Suppl. Figure 3: oxidative stress-induced cellular damage is not present in ipsilateral spinal cord dorsal horn of CPIP model rats. Suppl. Figure 4: evaluation of oxidative stress status in female CPIP model rats. Suppl. Figure 5: persistent EA treatment reduces overactivation of glial cells in SCDH of CPIP model rats. Suppl. Figure 6: persistent EA or NAC treatment reduces proinflammatory cytokine overexpression in hindpaw tissues of CPIP model rats. Suppl. Table 1: sequence of primers used for qPCR. Suppl. Table 2: complete list of statistical results (mean, SEM, SD, and confidence interval). Suppl. Table 3: expression changes of genes involved in oxidative stress, antioxidant defense, and reactive oxygen metabolism process.
