# Supplementary material for: Nrf2 Activation Mediates Antiallodynic Effect of Electroacupuncture on a Rat Model of Complex Regional Pain Syndrome Type-I through Reducing Local Oxidative Stress and Inflammation
Source: Oxid Med Cell Longev. 2022 Feb 14;2022:8035109. doi: 10.1155/2022/8035109 (PMC9054487; doi:10.1155/2022/8035109)
Supplement: Supplementary Materials — The supplementary materials contain the following figures and tables in one file: Suppl. Figure 1: original Western blot images. Suppl. Figure 2: high-quality hindpaw tissue RNA obtained for RNA-Seq. Suppl. Figure 3: oxidative stress-induced cellular damage is not present in ipsilateral spinal cord dorsal horn of CPIP model rats. Suppl. Figure 4: evaluation of oxidative stress status in female CPIP model rats. Suppl. Figure 5: persistent EA treatment reduces overactivation of glial cells in SCDH of CPIP model rats. Suppl. Figure 6: persistent EA or NAC treatment reduces proinflammatory cytokine overexpression in hindpaw tissues of CPIP model rats. Suppl. Table 1: sequence of primers used for qPCR. Suppl. Table 2: complete list of statistical results (mean, SEM, SD, and confidence interval). Suppl. Table 3: expression changes of genes involved in oxidative stress, antioxidant defense, and reactive oxygen metabolism process. [file 8035109.f1.zip › Suppl. Table 1.docx]

| Primers | Forward | Reverse | Amplicon size (bp) |
| --- | --- | --- | --- |
| β-actin | TGTCACCAACTGGGACGATA | GGGGTGTTGAAGGTCTCAAA | 165 |
| TNF-α | AAAGGACACCATGAGCACGGAAAG | CGCCACGAGCAGGAATGAGAAG | 136 |
| Il-1β | AACTGTGAAATAGCAGCTTTCG | CTGTGAGATTTGAAGCTGGATG | 138 |
| IL-6 | TGCACTGTCAGAAAACAATCTG | CCAGAGCAGATTTTCAATAGGC | 105 |

**Suppl. Table 1 Sequence of primers used for qPCR.**
