# Supplementary material for: Nrf2 Activation Mediates Antiallodynic Effect of Electroacupuncture on a Rat Model of Complex Regional Pain Syndrome Type-I through Reducing Local Oxidative Stress and Inflammation
Source: Oxid Med Cell Longev. 2022 Feb 14;2022:8035109. doi: 10.1155/2022/8035109 (PMC9054487; doi:10.1155/2022/8035109)
Supplement: Supplementary Materials — The supplementary materials contain the following figures and tables in one file: Suppl. Figure 1: original Western blot images. Suppl. Figure 2: high-quality hindpaw tissue RNA obtained for RNA-Seq. Suppl. Figure 3: oxidative stress-induced cellular damage is not present in ipsilateral spinal cord dorsal horn of CPIP model rats. Suppl. Figure 4: evaluation of oxidative stress status in female CPIP model rats. Suppl. Figure 5: persistent EA treatment reduces overactivation of glial cells in SCDH of CPIP model rats. Suppl. Figure 6: persistent EA or NAC treatment reduces proinflammatory cytokine overexpression in hindpaw tissues of CPIP model rats. Suppl. Table 1: sequence of primers used for qPCR. Suppl. Table 2: complete list of statistical results (mean, SEM, SD, and confidence interval). Suppl. Table 3: expression changes of genes involved in oxidative stress, antioxidant defense, and reactive oxygen metabolism process. [file 8035109.f1.zip › Suppl. Table 2 Statistic dataset OMCL.pdf]

Figure1	E		Control	CPIP
	Normalized AUC(%)	Mean	100	34.32
	Ipsilateral	Std. Deviation	12.11	17.71
		Std. Error of Mean	4.944	7.234
		Lower 95% CI of mean	78.01	19.29
		Upper 95% CI of mean	122	49.36
	G		Control	CPIP
	Normalized AUC(%)	Mean	100	53.67
	Contralateral	Std. Deviation	2.282	10.34
		Std. Error of Mean	0.932	4.221
		Lower 95% CI of mean	97.17	40.84
		Upper 95% CI of mean	102.8	66.51

Figure3	A		Control	3d	7d	10d	
	SOD	Mean	5.802	4.113	4.179	4.885	
		Std. Deviation	0.7837	0.1782	0.2664	0.9994	
		Std. Error of Mean	0.3505	0.0797	0.1191	0.447	
		95% CI of mean	.828-6.77	3.834-4.393	3.992-4.366	3.836-5.933	
	B		Control	3d	7d	10d	
	GSH	Mean	14.87	4.156	6.11	6.741	
		Std. Deviation	2.733	2.54	1.228	4.696	
		Std. Error of Mean	1.116	1.037	0.5014	1.917	
		95% CI of mean	12-17.74	1.491-6.822	4.821-7.398	1.813-11.67	
	C		Control	3d	7d	10d	
	MDA	Mean	5.289	10.06	10.35	11.28	
		Std. Deviation	2.057	0.7162	2.231	1.081	
		Std. Error of Mean	0.839767	0.3203	0.9976	0.4834	
		Lower 95% CI of mean	2.519	8.718	7.791	10.39	
		Upper 95% CI of mean	8.059	11.4	12.9	12.17	
	E	Mean	100	118.2	209.4	154.2	
	4HNE	Std. Deviation	56.34	58.79	59.19	68.78	
		Std. Error of Mean	21.3	22.22	22.37	26	
		95% CI of mean	7.89-152.	63.82-172.6	154.6-264.1	90.58-217.8	
	F		Control	7d			
	SOD	Mean	5.733	4.861			
		Std. Deviation	1.24	0.9036			
		Std. Error of Mean	0.5547	0.369			
		Lower 95% CI of mean	4.193	3.739			
		Upper 95% CI of mean	7.273	5.983			
			Control	7d			
	G	Mean	6.731	5.734			
	GSH	Std. Deviation	4.085	2.114			
		Std. Error of Mean	1.827	0.9456			
		Lower 95% CI of mean	4.106	0.6619			
		Upper 95% CI of mean	9.357	10.81			
	H		Control	7d			
	MDA	Mean	11	4.779			
		Std. Deviation	4.736	1.389			
		Std. Error of Mean	2.118	0.621			
		Lower 95% CI of mean	5.117	3.054			
		Upper 95% CI of mean	16.88	6.503			
	J		Control	3d	7d	10d	14d
	4HNE	Mean	100	87.29	112.1	93.25	118.4
		Std. Deviation	13.86	18.6	39.6	30.91	30.69
		Std. Error of Mean	6.197	8.316	17.71	13.82	13.73
		95% CI of mean	2.79-117.	64.2-110.4	62.95-161.3	54.87-131.6	80.33-156.5
	K		Control	3d	7d	10d	
	SOD	Mean	116.8	114.1	121.2	110.6	
		Std. Deviation	2.278	4.14	3.976	3.366	
		Std. Error of Mean	0.93	1.69	1.623	1.374	
		Lower 95% CI of mean	114.4	109	116.3	106.4	
		Upper 95% CI of mean	119.1	119.3	126.1	114.8	
	L		Control	3d	7d	10d	
	GSH	Mean	182.5	179.3	187.9	176.3	
		Std. Deviation	16.26	11.43	22.75	22.82	
		Std. Error of Mean	6.637	4.665	9.286	9.315	
		95% CI of mean	65.5-199.	165.1-193.5	159.7-216.2	147.9-204.6	
	M		Control	3d	7d	10d	
	MDA	Mean	47.63	38.2	44.92	61.28	
		Std. Deviation	3.518	10.05	10.35	17.98	
		Std. Error of Mean	1.573	4.495	4.628	8.039	
		95% CI of mean	3.26-51.9	25.72-50.68	32.07-57.77	38.96-83.61	

Figue 4	C		Con +Veh	CPIP +Veh	CPIP+NAC
	Normalized AUC(%)	Mean	100	44.36	82.22
	Ipsilateral	Std. Deviation	5.917	3.937	11.69
		Std. Error of Mean	2.646	1.761	5.226
		95% CI of mean	92.65-107.3	39.48-49.25	67.71-96.73
	E	c-Fos	Contl	CPIP+Veh	CPIP+NAC
	c-Fos cell counts/field	Mean	7.333	33.5	9
		Std. Deviation	6.218	5.718	6.164
		Std. Error of Mean	2.539	2.335	2.517
		Lower 95% CI of mean	0.8077	27.5	2.531
		Upper 95% CI of mean	13.86	39.5	15.47
	G	GFAP	Contl	CPIP+Veh	CPIP+NAC
	Normalized fluorescence intensity/fi	Mean	100	155.7	83.14
		Std. Deviation	5.842	33.29	10.75
		Std. Error of Mean	2.613	14.89	4.807
		Lower 95% CI of mean	92.75	114.4	69.8
		Upper 95% CI of mean	107.3	197	96.49
	H	GFAP	Contl	CPIP+Veh	CPIP+NAC
	Percentage of GFAP area	Mean	0.3017	3.222	0.3829
		Std. Deviation	0.2031	0.9705	0.2101
		Std. Error of Mean	0.09083	0.434	0.09396
		Lower 95% CI of mean	0.0495	2.017	0.1221
		Upper 95% CI of mean	0.5539	4.427	0.6438
	J	OX42	Contl	CPIP+Veh	CPIP+NAC
	Normalized fluorescence intensity/fi	Mean	100	148.3	92.79
		Std. Deviation	22.07	34.87	18.29
		Std. Error of Mean	9.869	15.59	8.18
		Lower 95% CI of mean	72.6	105	70.08
		Upper 95% CI of mean	127.4	191.6	115.5
	K	OX42	Contl	CPIP+Veh	CPIP+NAC
	Percentage of OX42 area	Mean	0.6255	4.259	0.5218
		Std. Deviation	0.1928	1.297	0.1847
		Std. Error of Mean	0.08622	0.5799	0.0826
		Lower 95% CI of mean	0.3862	2.649	0.2925
		Upper 95% CI of mean	0.8649	5.869	0.7511

Figure 5				
	C		Con +Veh	Con+H_2O_2
	Normalized AUC(%)	Mean	100	39.12
	Ipsilateral	Std. Deviation	9.385	6.004
		Std. Error of Mean	3.831	2.451
		95% CI of mean	88.35-111.7	31.66-46.57
	E	c-Fos	Con +Veh	Con+H_2O_2
	c-Fos cell counts/field	Mean	6.833	19
		Std. Deviation	3.371	3.033
		Std. Error of Mean	1.376	1.238
		Lower 95% CI of mean	3.295	15.82
		Upper 95% CI of mean	10.37	22.18
	G	GFAP	Con +Veh	Con+H_2O_2
	Normalized fluorescence intensity/field(	Mean	100	167.6
		Std. Deviation	8.089	39.3
		Std. Error of Mean	3.302	16.042
		Lower 95% CI of mean	89.96	118.8
		Upper 95% CI of mean	110	216.4
	H	GFAP	Con +Veh	Con+H_2O_2
	Percentage of GFAP area	Mean	0.807	3.187
		Std. Deviation	0.2716	0.4691
		Std. Error of Mean	0.111	0.191
		Lower 95% CI of mean	0.4698	2.605
		Upper 95% CI of mean	1.144	3.77
	J	OX42	Con +Veh	Con+H_2O_2
	Normalized fluorescence intensity/field(	Mean	100	148.8
		Std. Deviation	10.73	19.57
		Std. Error of Mean	4.379	7.988
		Lower 95% CI of mean	86.68	124.5
		Upper 95% CI of mean	113.3	173.1
	K	OX42	Con +Veh	Con+H_2O_2
	Percentage of OX42 area	Mean	0.6282	4.673
		Std. Deviation	0.3369	0.9585
		Std. Error of Mean	0.138	0.391
		Lower 95% CI of mean	0.2098	3.482
		Upper 95% CI of mean	1.047	5.863

				Con	CPIP	EA	Sham EA
Figure 6	Figure6	C		Con	CPIP	EA	Sham EA
		Normalized AUC(%)	Mean	100	45.95	86.36	48.08
		Ipsilateral	Std. Deviation	5.93	2.752	10.49	3.757
			Std. Error of Mean	2.421	1.123	4.283	1.534
			Lower 95% CI of mean	97.45	45.47	76.66	48.6
			Upper 95% CI of mean	102.5	56.44	96.06	55.55
		E		Con	CPIP	EA	Sham EA
		Normalized AUC(%)	Mean	100	50.95	86.36	62.08
		Contralateral	Std. Deviation	2.752	5.93	3.757	10.49
			Std. Error of Mean	1.123	2.421	1.534	4.283
			Lower 95% CI of mean	97.45	45.47	76.66	48.6
			Upper 95% CI of mean	102.5	56.44	96.06	55.55

Figure 7

			Con	CPIP	EA	Sham EA
A	SOD	Mean	7.021	4.082	6.477	3.915
		Std. Deviation	3.649	0.6167	2.943	0.5274
		Std. Error of Mea	1.490	0.252	1.201	0.215
		Lower 95% CI of me	4.816	3.709	4.698	3.596
		Upper 95% CI of me	9.226	4.455	8.256	4.233
B			Con	CPIP	EA	Sham EA
	GSh	Mean	10.72	6.378	9.197	6.029
		Std. Deviation	3.51	0.9805	2.728	0.5848
		Std. Error of Mea	1.433	0.400	1.114	0.239
		Lower 95% CI of me	8.02	5.624	7.1	5.579
		Upper 95% CI of me	13.41	7.132	11.29	6.478
C			Con	CPIP	EA	Sham EA
	MDA	Mean	8.032	16.21	5.842	12.32
		Std. Deviation	1.834	6.674	1.486	5.455
		Std. Error of Mea	0.749	2.725	0.607	2.227
		Lower 95% CI of me	6.498	10.04	4.467	7.273
		Upper 95% CI of me	9.565	22.38	7.216	17.36
D			Con	CPIP	EA	Sham EA
	H_2O_2	Mean	5.448	8.389	4.764	7.583
		Std. Deviation	2.591	0.8625	1.704	1.182
		Std. Error of Mea	1.058	0.352	0.696	0.483
		Lower 95% CI of me	3.053	7.591	2.976	6.343
		Upper 95% CI of me	7.844	9.187	6.551	8.823
F			Con	CPIP	EA	Sham EA
	Normalized fluorescence intensity/field(%	Mean	100	215.7	105.6	176.8
		Std. Deviation	32.15	61.5	42.01	35.81
		Std. Error of Me	13.125	25.107	17.151	14.619
		Lower 95% CI of	72.01	175.8	88.44	138.2
		Upper 95% CI of	128	235.7	122.7	175.5
G		4HNE	Con	CPIP	EA	Sham EA
	Normalized ratio to β-actin(%)	Mean	100	195.6	112.3	201.9
		Std. Deviation	60.31	51.64	46.11	68.05
		Std. Error of Mea	24.621	21.082	18.824	27.781
		95% CI of mean	[illegible]4.23-155.[illegible]	[illegible]47.8-243.	9.64-154.	139-264.8

Figure 8	B	NRF2	Con	CPIP	EA	Sham EA
Normalized ratio to β-actin(%		Mean	100	34.07	73.33	122.2
		Std. Deviation	37.85	19.72	40.4	20.69
		Std. Error of Mean	14.306	8.819	16.493	9.253
		Lower 95% CI of mean	58.09	2.683	27.03	70.8
		Upper 95% CI of mean	152.1	65.45	173.7	173.6
	C		CPIP+EA+Veh	CPIP+EA+ML385		
	SOD	Mean	5.265	3.834		
		Std. Deviation	0.8497	0.6329		
		Std. Error of Mean	0.347	0.258		
		Lower 95% CI of mean	4.612	3.348		
		Upper 95% CI of mean	5.918	4.321		
	D		CPIP+EA+Veh	CPIP+EA+ML385		
	GSH	Mean	6.184	1.954		
		Std. Deviation	4.255	2.89		
		Std. Error of Mean	1.737	1.180		
		Lower 95% CI of mean	2.913	-0.2677		
		Upper 95% CI of mean	9.455	4.175		
	E		CPIP+EA+Veh	CPIP+EA+ML385		
	H2O2	Mean	2.308	3.985		
		Std. Deviation	0.95	0.8078		
		Std. Error of Mean	0.388	0.330		
		Lower 95% CI of mean	1.311	3.138		
		Upper 95% CI of mean	3.304	4.833		
	F		CPIP+EA+Veh	CPIP+EA+ML385		
	MDA	Mean	3.583	8.837		
		Std. Deviation	3.103	3.838		
		Std. Error of Mean	1.267	1.567		
		Lower 95% CI of mean	0.989	5.629		
		Upper 95% CI of mean	6.177	12.05		
	H		Control+Veh	CPIP+EA+Veh	CPIP+EA+ML385	
	Normalized AUC(%)	Mean	100	82.54	44.75	
	Ipsilateral	Std. Deviation	5.075	12.59	8.748	
		Std. Error of Mean	2.072	5.140	3.571	
		Lower 95% CI of mean	94.67	72.86	38.03	
		Upper 95% CI of mean	105.3	92.22	51.47	

Figure 9

			Con	CPIP	EA	NAC
B	%H_2O_2 positive responding neuron	Mean	25.98	44.34	26.84	27.91
		Std. Deviation	5.886	7.473	5.44	3.407
		Std. Error of Mean	2.943	3.737	2.720	1.704
		95% CI of mean	21.08-30.89	38.09-50.58	22.29-31.39	25.06-30.76
D	Δchange in R340/380	Mean	0.4399	1.178	0.3849	0.3315
		Std. Deviation	0.4983	0.6634	0.5173	0.5552
		Std. Error of Mean	0.288	0.383	0.299	0.321
		95% CI of mean	0.3411-0.5388	1.047-1.31	0.2823-0.4875	0.2214-0.4417

Figure 1(	B	0d	Contl	CPIP	CPIP+EA	2.23607
Normalized regional blood flow(%)		Mean	100	97.56	87.61	
		Std. Deviation	8.94	9.734	2.925	
		Std. Error of Mear	3.998	4.353	1.308	
		Lower 95% CI of mea	85.78	82.07	82.95	
		Upper 95% CI of mea	114.2	113	92.26	
		1d	Contl	CPIP	CPIP+EA	
		Mean	100	56.49	65.88	
		Std. Deviation	6.639	6.513	9.477	
		Std. Error of Mear	2.969	2.913	4.238	
		Lower 95% CI of mea	89.44	46.12	50.8	
		Upper 95% CI of mea	110.6	66.85	80.96	
		3d	Contl	CPIP	CPIP+EA	
		Mean	100	53.38	101.8	
		Std. Deviation	12.71	9.174	9.763	
		Std. Error of Mear	5.684	4.103	4.366	
		Lower 95% CI of mea	79.78	38.78	86.22	
		Upper 95% CI of mea	120.2	67.97	117.3	
		5d	Contl	CPIP	CPIP+EA	
		Mean	100	69.93	101.3	
		Std. Deviation	11.08	16.93	6.843	
		Std. Error of Mear	4.955	7.571	3.060	
		Lower 95% CI of mea	82.37	42.99	90.39	
		Upper 95% CI of mea	117.6	96.88	112.2	
		7d	Contl	CPIP	CPIP+EA	
		Mean	100	70.35	117.1	
		Std. Deviation	15.61	12.08	10.57	
		Std. Error of Mear	6.981	5.402	4.727	
		Lower 95% CI of mea	75.15	51.13	100.3	
		Upper 95% CI of mea	124.8	89.58	133.9	
	D		Con +Veh	CPIP +Veh	CPIP+NAC	
Normalized regional blood flow(%)		Mean	100	63	71.91	
		Std. Deviation	13.3	5.687	8.023	
		Std. Error of Mear	5.028	2.149	3.033	
		Lower 95% CI of mea	87.7	57.74	64.49	
		Upper 95% CI of mea	112.3	68.26	79.33	
