# Supplementary material for: Nrf2 Activation Mediates Antiallodynic Effect of Electroacupuncture on a Rat Model of Complex Regional Pain Syndrome Type-I through Reducing Local Oxidative Stress and Inflammation
Source: Oxid Med Cell Longev. 2022 Feb 14;2022:8035109. doi: 10.1155/2022/8035109 (PMC9054487; doi:10.1155/2022/8035109)
Supplement: Supplementary Materials — The supplementary materials contain the following figures and tables in one file: Suppl. Figure 1: original Western blot images. Suppl. Figure 2: high-quality hindpaw tissue RNA obtained for RNA-Seq. Suppl. Figure 3: oxidative stress-induced cellular damage is not present in ipsilateral spinal cord dorsal horn of CPIP model rats. Suppl. Figure 4: evaluation of oxidative stress status in female CPIP model rats. Suppl. Figure 5: persistent EA treatment reduces overactivation of glial cells in SCDH of CPIP model rats. Suppl. Figure 6: persistent EA or NAC treatment reduces proinflammatory cytokine overexpression in hindpaw tissues of CPIP model rats. Suppl. Table 1: sequence of primers used for qPCR. Suppl. Table 2: complete list of statistical results (mean, SEM, SD, and confidence interval). Suppl. Table 3: expression changes of genes involved in oxidative stress, antioxidant defense, and reactive oxygen metabolism process. [file 8035109.f1.zip › Suppl. Table 3 ______.pdf]

Suppl. Table 3. Expression changes of genes involved in oxidative stress, antioxidant defense and reactive oxygen metabolism process.

Genes	Fold changes	q-value
Glutathione peroxidases		
Gpx1	0.22	3.20E-45
Gpx2	-1.30	4.74E-39
Gpx3	1.10	0
Gpx4	0.14	1.09E-11
Gpx7	-0.18	2.98E-07
Peroxiredoxins		
Gstk1	0.05	0.20
Ehd2	0.19	1.23E-32
Prdx1	0.02	0.19
Prdx2	0.06	-0.01
Prdx3	0.19	1.63E-05
Prdx4	-0.02	0.16
Prdx5	0.13	7.84E-06
Prdx6	0.30	2.33E-18
Other peroxidases		
Aass	0.62	0.03
Apc	0.22	3.70E-11
Cat	0.44	1.33E-64
Duox1	0.50	8.44E-15
Epx	1.41	0.01
Kif9	0.38	0.10
Lpo	-0.37	0.09
Mpo	1.02	0.03
Ptgs1	0.03	0.23
Ptgs2	0.30	0.06
Rag2	0.59	0.21
Slc41a3	0.07	0.16
Tmod1	0.60	1.87E-76
Other antioxidants		
Gsr	0.18	7.14E-05
Nxn	0.11	0.004
Srxn1	-0.32	3.32E-07
Txnrd1	0.24	6.04E-10
Txnrd2	0.19	0.02
Txnrd3	-0.06	0.15
Superoxide dismutases		
Sod1	0.15	8.28E-09
Sod2	0.11	5.39E-05
Sod3	0.44	0
Genes involved in superoxide metabolism		
Ccs	0.11	0.03
Cyba	0.40	5.91E-34
Ncf1	0.59	8.51E-53
Ncf4	0.66	3.48E-33
Ncf2	0.55	4.82E-07
Nos1	0.22	0.04
Nox4	0.96	6.08E-17
Noxa1	-4.57	0.0004
Noxo1	1.19	0.03
Recql4	-0.22	0.01

Genes	Fold changes	q-value
Genes involved in ROS metabolism		
Fmo2	0.69	1.49E-47
Other oxidative stress responsive genes		
Als2	0.27	2.70E-06
Apoe	0.56	0
Ercc2	0.15	0.01
Ercc6	0.11	0.04
Gab1	0.40	9.25E-29
Idh1	0.29	2.67E-16
Mpp4	0.26	0.22
Nqo1	-0.51	2.82E-20
Nudtl5	1.05	7.23E-05
Park7	0.19	6.14E-09
Ppp1r15b	0.07	0.03
Prnp	0.10	2.38E-08
Psmb5	0.12	5.28E-05
Txnip	0.15	3.07E-64
Ucp3	-0.11	0.01
Xpa	0.49	6.96E-05
Oxygen transporters		
Aqr	0.27	1.16E-09
Cygb	0.63	3.60E-237
Dnm2	0.31	4.98E-37
Fancc	-0.16	0.14
Ift172	0.40	1.14E-16
Mb	-0.02	0.03
Ngb	0.18	0.27
Slc38al	-0.31	0.0002
Vim	0.51	0
