# Supplementary material for: Nrf2 Activation Mediates Antiallodynic Effect of Electroacupuncture on a Rat Model of Complex Regional Pain Syndrome Type-I through Reducing Local Oxidative Stress and Inflammation
Source: Oxid Med Cell Longev. 2022 Feb 14;2022:8035109. doi: 10.1155/2022/8035109 (PMC9054487; doi:10.1155/2022/8035109)
Supplement: Supplementary Materials — The supplementary materials contain the following figures and tables in one file: Suppl. Figure 1: original Western blot images. Suppl. Figure 2: high-quality hindpaw tissue RNA obtained for RNA-Seq. Suppl. Figure 3: oxidative stress-induced cellular damage is not present in ipsilateral spinal cord dorsal horn of CPIP model rats. Suppl. Figure 4: evaluation of oxidative stress status in female CPIP model rats. Suppl. Figure 5: persistent EA treatment reduces overactivation of glial cells in SCDH of CPIP model rats. Suppl. Figure 6: persistent EA or NAC treatment reduces proinflammatory cytokine overexpression in hindpaw tissues of CPIP model rats. Suppl. Table 1: sequence of primers used for qPCR. Suppl. Table 2: complete list of statistical results (mean, SEM, SD, and confidence interval). Suppl. Table 3: expression changes of genes involved in oxidative stress, antioxidant defense, and reactive oxygen metabolism process. [file 8035109.f1.zip › Supplementary figures tables and legends Li XJ.docx]

**Supplementary Materials**

**Suppl. Figure 1 Original images of Western blot in this study.**

**Suppl. Figure 2. High quality hind paw tissue RNA obtained for RNA-Seq.** (A-F) RNA integrity number (RIN) determined by TapeStation from control and CPIP model groups.

**Suppl. Figure 3. Oxidative stress-induced cellular damage is not present in ipsilateral spinal cord dorsal horn of CPIP model rats.** (A) Representative pictures showing 8-OHG immunostaining (red), a marker for oxidative damage in cellular nucleic acids, in ipsilateral SCDH of control and CPIP model rats 3, 7, and 10 d after model establishment. DAPI was used for labeling all cells (blue). (B&C) Summarized data of 8-OHG positively labeled cells (B) and normalized fluorescence intensity of 8-OHG staining (C) per observation field in each group. n = 5-6 rats/group.

**Suppl. Figure 4. Evaluation of oxidative stress status in female CPIP model rats.** (A-D) Results showing SOD activity (A), GSH-Px activity (B), H_2_O_2_ (C) and MDA content (D) determined in ipsilateral hind paw tissues from female control and female CPIP model rats 7 d after model establishment. (E-H) Results showing SOD activity (E), GSH-Px activity (F), H_2_O_2_ (G) and MDA content (H) determined in ipsilateral spinal cord from female control and female CPIP model rats 7 d after model establishment. n = 6 rats/group. ^**^p < 0.01, ^*^p < 0.05 *vs*. Control group. (I) Time course of 50% PWT changes after NAC/vehicle treatment in female rats. CPIP+NAC group receives daily NAC (200 mg/kg, i.p.) treatment, whereas CPIP+Veh group receives vehicle (PBS, i.p.) treatment. (J) Summary of AUC as in (I). n = 6 rats/group. ^**^p < 0.01 *vs*. Control+Veh group, ^##^p < 0.01 *vs*. CPIP+Veh group. Student’s *t* test was used for statistical analysis in panels (A-H). Two-way ANOVA followed by Tukey post-hoc test was used for statistical analysis in panel (I). One-way ANOVA followed by Tukey post-hoc test was used for statistical analysis in panel (J).

**Suppl. Figure 5. Persistent EA treatment reduces over-activation of glial cells in SCDH of CPIP model rats. (A)** Representative pictures showing the astrocytic marker GFAP staining from Control, CPIP, CPIP+EA and CPIP+Sham EA group. (B&C) Summary of the normalized fluorescence intensity (B) and percentage (C) of GFAP staining per observation field. (D) Representative pictures showing the microglial marker OX42 staining from Control, CPIP, CPIP+EA and CPIP+Sham EA group. (E&F) Summary of the normalized fluorescence intensity (E) and percentage (F) of OX42 staining per observation field. n = 5 rats/group. ^**^p < 0.01 *vs*. Control group. ^##^p < 0.01, ^#^p < 0.05 *vs*. CPIP+Sham EA group. One-way ANOVA followed by Tukey post-hoc test was used for statistical analysis.

**Suppl. Figure 6. Persistent EA or NAC treatment reduces pro-inflammatory cytokine over-expression in hind paw tissues of CPIP model rats.** (A-C) qPCR determination of mRNA expression of some representative pro-inflammatory cytokines, including *Tnf-α* (A), *Il-1β* (B) and *Il-6* (C) in hind paw tissues of control, CPIP, CPIP+EA and CPIP+NAC groups, 7 d after model establishment. n = 6 rats/group. ^**^p < 0.01, ^*^p < 0.05. One-way ANOVA followed by Tukey post-hoc test was used for statistical analysis.

**Suppl. Table 1. Sequences of the qPCR primers.**

**Suppl. Table 2. Complete list of statistical results (mean, SEM, SD and confidence interval) in this study.**

| **Primers** | **Forward** | **Reverse** | **Amplicon size (bp)** |
| --- | --- | --- | --- |
| *β-actin* | TGTCACCAACTGGGACGATA | GGGGTGTTGAAGGTCTCAAA | 165 |
| *Tnf-α* | AAAGGACACCATGAGCACGGAAAG | CGCCACGAGCAGGAATGAGAAG | 136 |
| *Il-1β* | AACTGTGAAATAGCAGCTTTCG | CTGTGAGATTTGAAGCTGGATG | 138 |
| *Il-6* | TGCACTGTCAGAAAACAATCTG | CCAGAGCAGATTTTCAATAGGC | 105 |

**Suppl. Table 3. Summary and functional classification of genes involved in oxidative stress, antioxidant defense and reactive oxygen metabolism process.** Up-regulated DEGs are displayed in red, whereas down-regulated DEGs are in blue color. Non-DEGs are in black color.
