# Supplementary figures and images for: Nrf2 Activation Mediates Antiallodynic Effect of Electroacupuncture on a Rat Model of Complex Regional Pain Syndrome Type-I through Reducing Local Oxidative Stress and Inflammation
Source: Oxid Med Cell Longev. 2022 Feb 14;2022:8035109. doi: 10.1155/2022/8035109 (PMC9054487; doi:10.1155/2022/8035109)

Figure3

D Ipsilateral skin

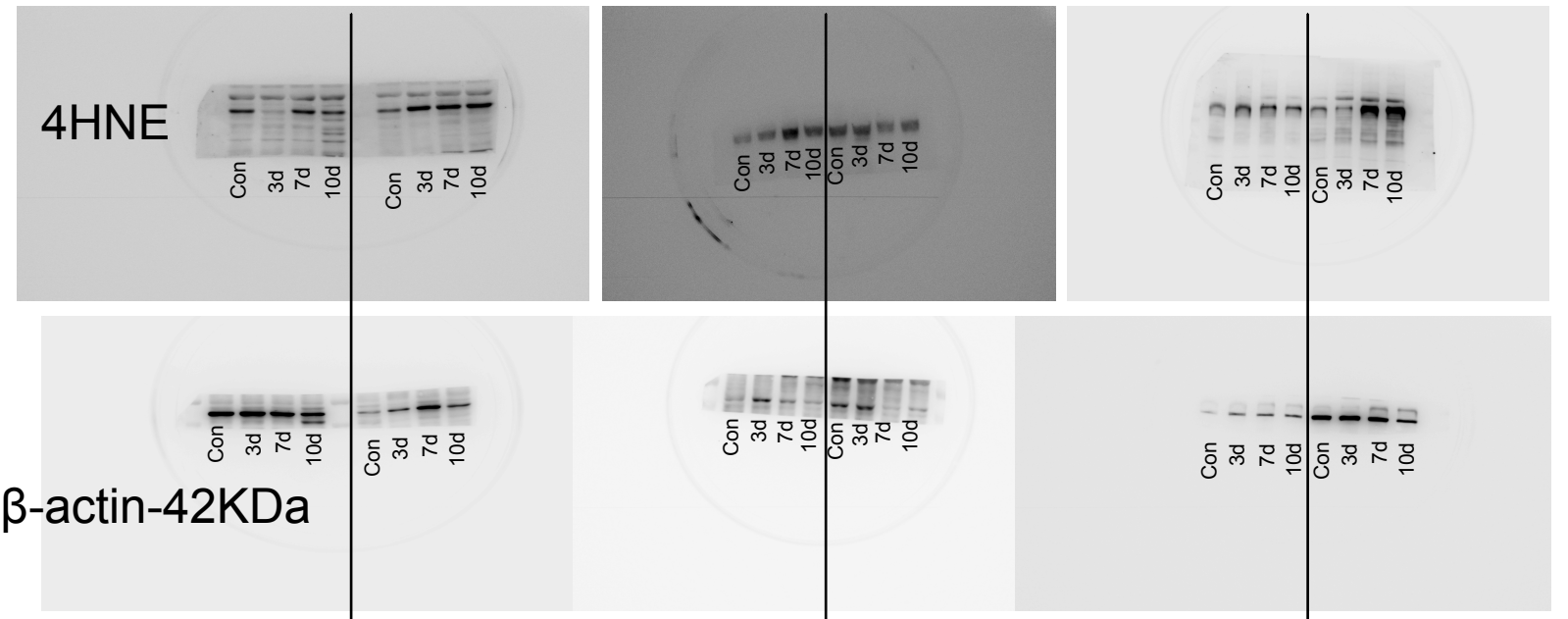

I Ipsilateral spinal cord

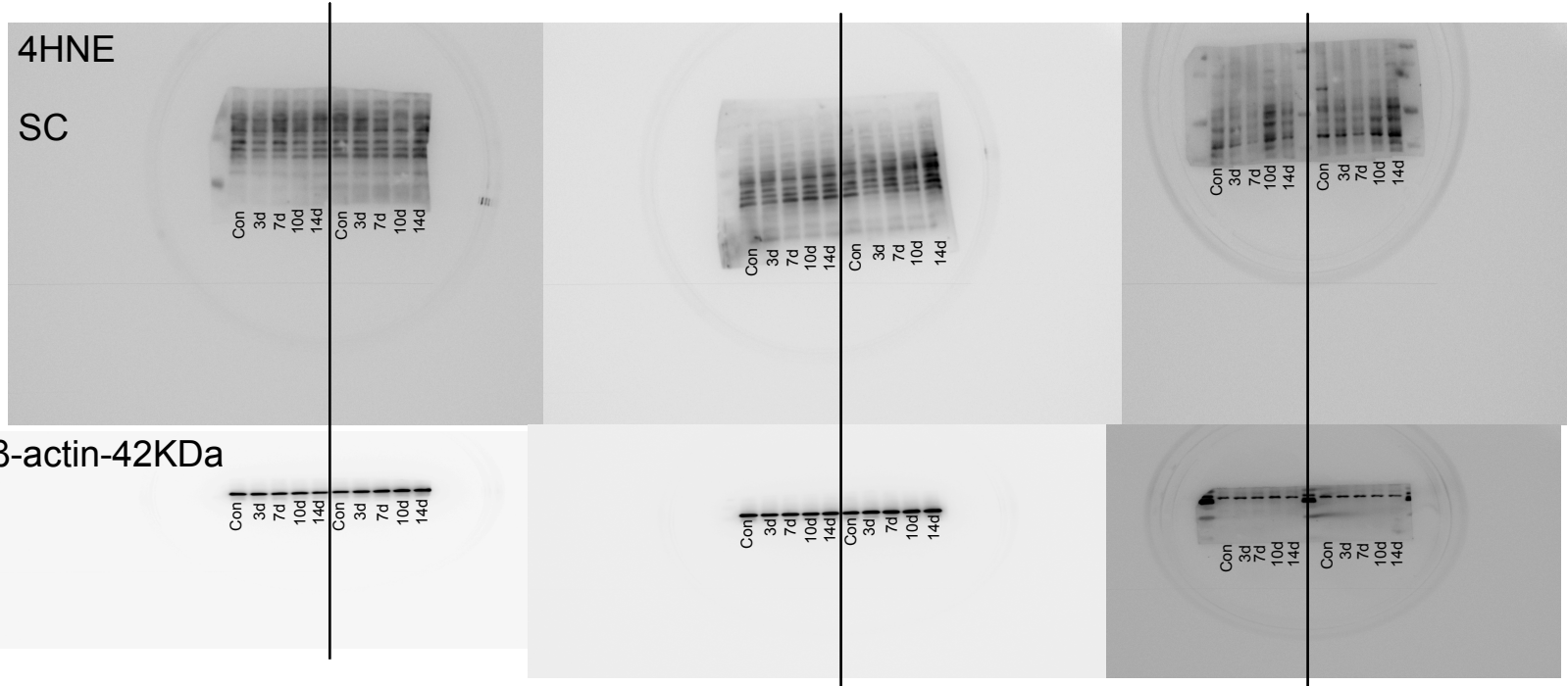

Figure 7

G

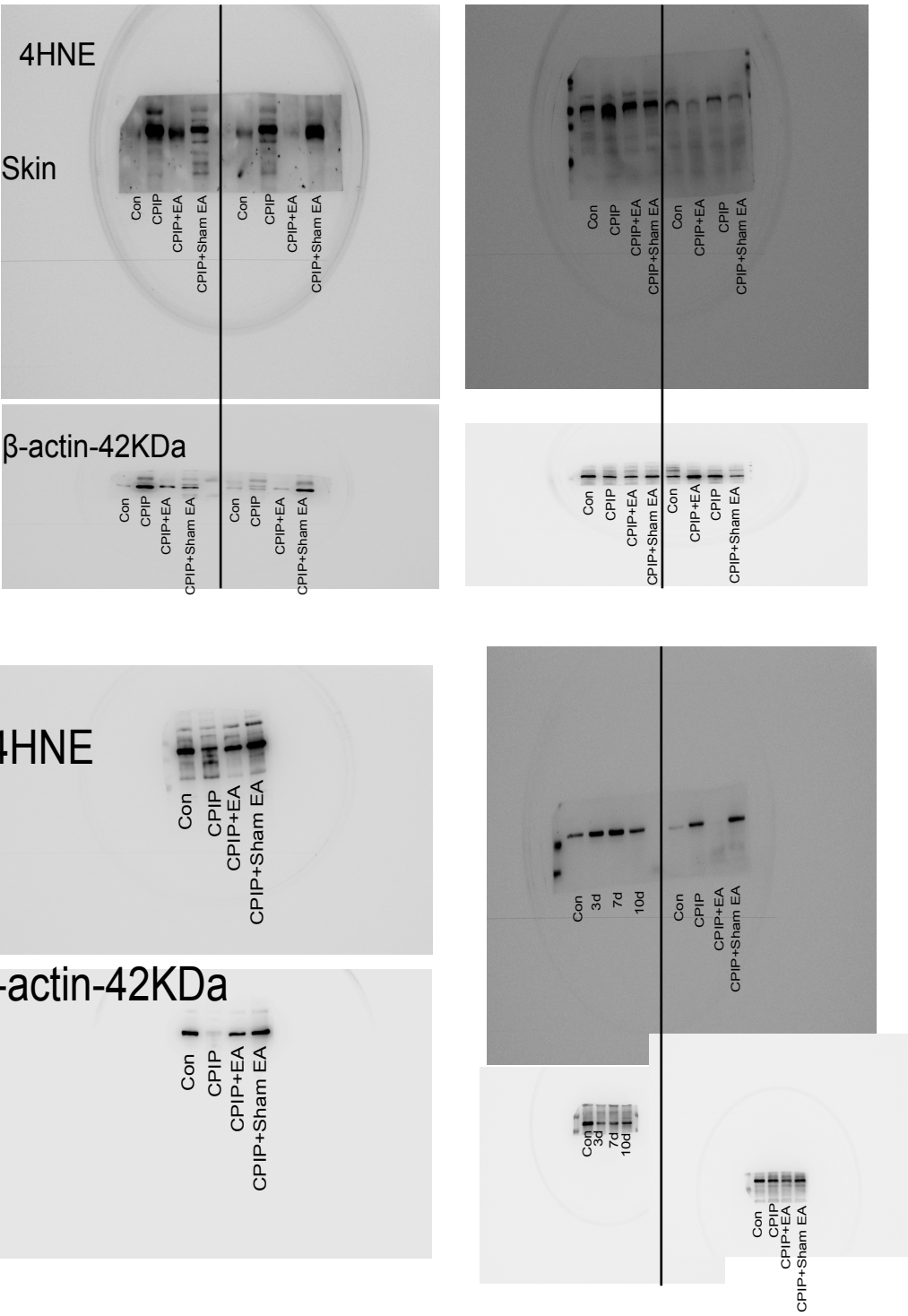

Figure 8

A

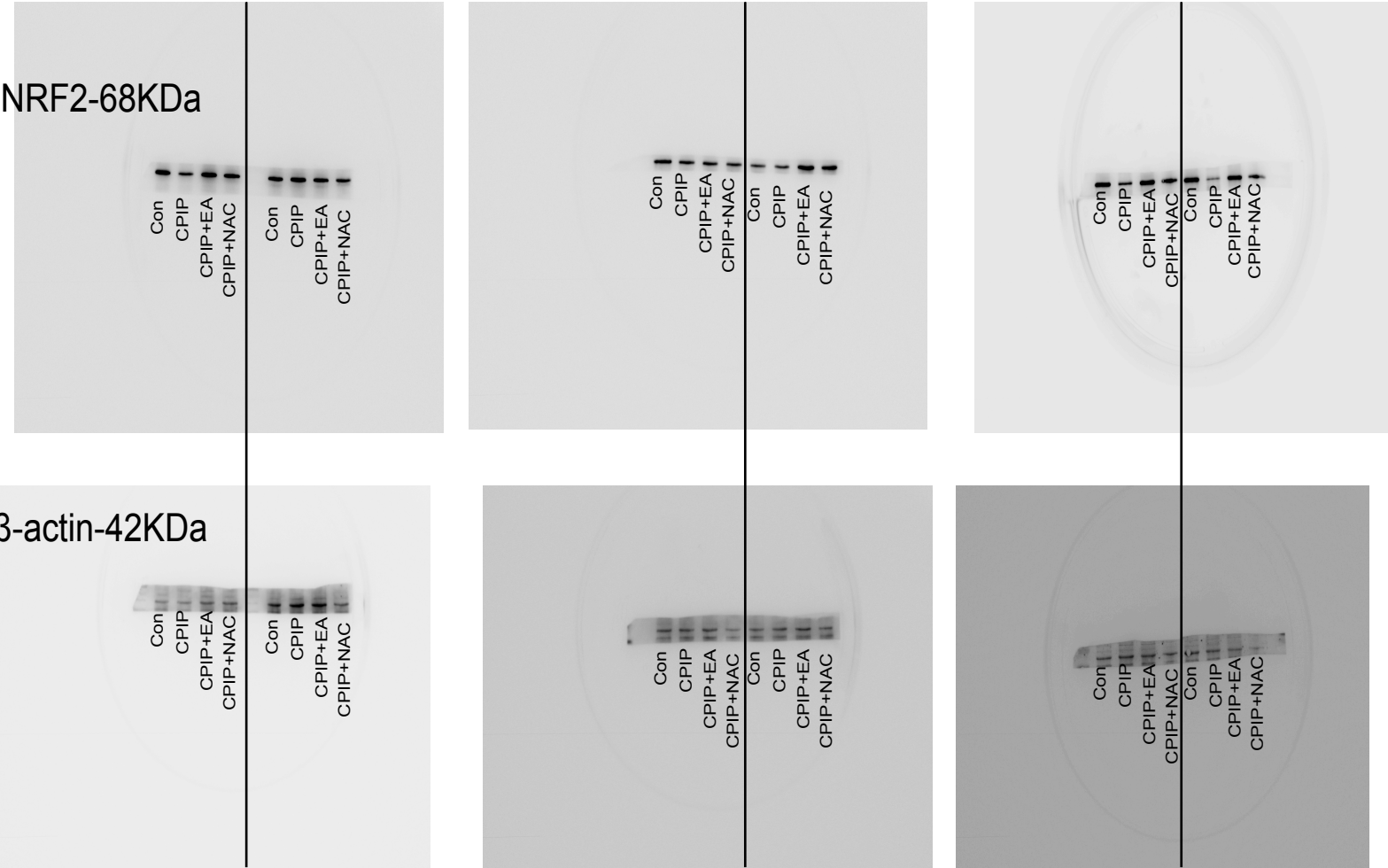

Supplement: Supplementary Materials — The supplementary materials contain the following figures and tables in one file: Suppl. Figure 1: original Western blot images. Suppl. Figure 2: high-quality hindpaw tissue RNA obtained for RNA-Seq. Suppl. Figure 3: oxidative stress-induced cellular damage is not present in ipsilateral spinal cord dorsal horn of CPIP model rats. Suppl. Figure 4: evaluation of oxidative stress status in female CPIP model rats. Suppl. Figure 5: persistent EA treatment reduces overactivation of glial cells in SCDH of CPIP model rats. Suppl. Figure 6: persistent EA or NAC treatment reduces proinflammatory cytokine overexpression in hindpaw tissues of CPIP model rats. Suppl. Table 1: sequence of primers used for qPCR. Suppl. Table 2: complete list of statistical results (mean, SEM, SD, and confidence interval). Suppl. Table 3: expression changes of genes involved in oxidative stress, antioxidant defense, and reactive oxygen metabolism process. [file 8035109.f1.zip › Suppl. Fig1 WB.pdf]

Ipsilateral skin

**A**

Sham 1

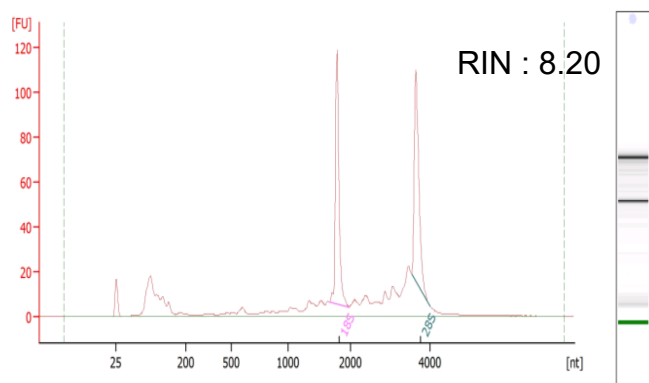

**D**

Model 1

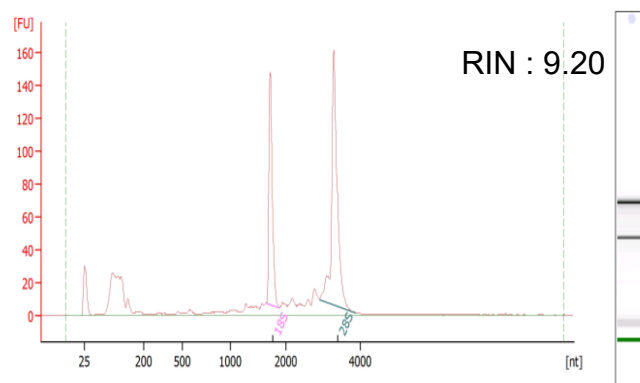

**B**

Sham 2

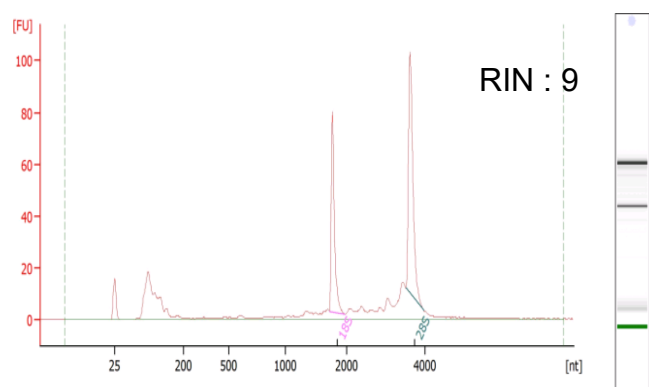

**E**

Model 2

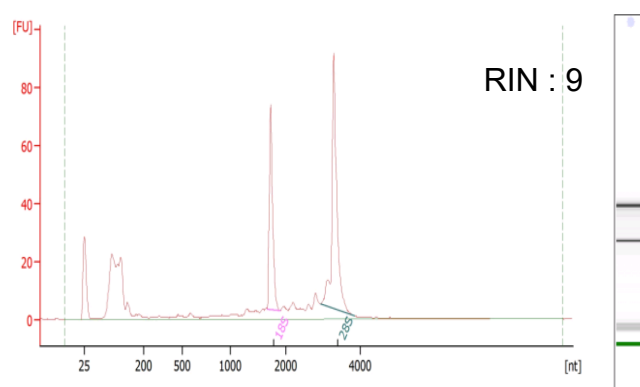

**C**

Sham 3

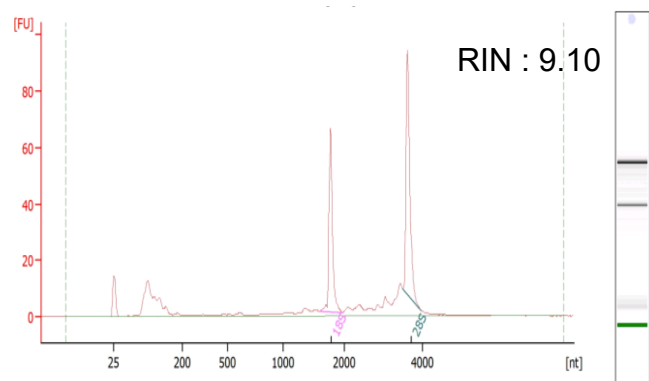

**F**

Model 3

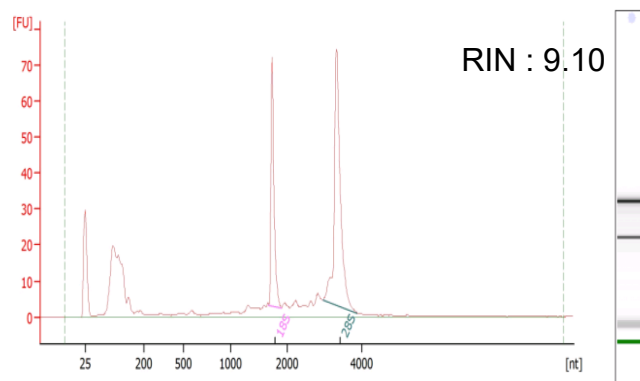

Supplement: Supplementary Materials — The supplementary materials contain the following figures and tables in one file: Suppl. Figure 1: original Western blot images. Suppl. Figure 2: high-quality hindpaw tissue RNA obtained for RNA-Seq. Suppl. Figure 3: oxidative stress-induced cellular damage is not present in ipsilateral spinal cord dorsal horn of CPIP model rats. Suppl. Figure 4: evaluation of oxidative stress status in female CPIP model rats. Suppl. Figure 5: persistent EA treatment reduces overactivation of glial cells in SCDH of CPIP model rats. Suppl. Figure 6: persistent EA or NAC treatment reduces proinflammatory cytokine overexpression in hindpaw tissues of CPIP model rats. Suppl. Table 1: sequence of primers used for qPCR. Suppl. Table 2: complete list of statistical results (mean, SEM, SD, and confidence interval). Suppl. Table 3: expression changes of genes involved in oxidative stress, antioxidant defense, and reactive oxygen metabolism process. [file 8035109.f1.zip › Suppl. Fig2 skin RNA.pdf]

Suppl. Figure 3

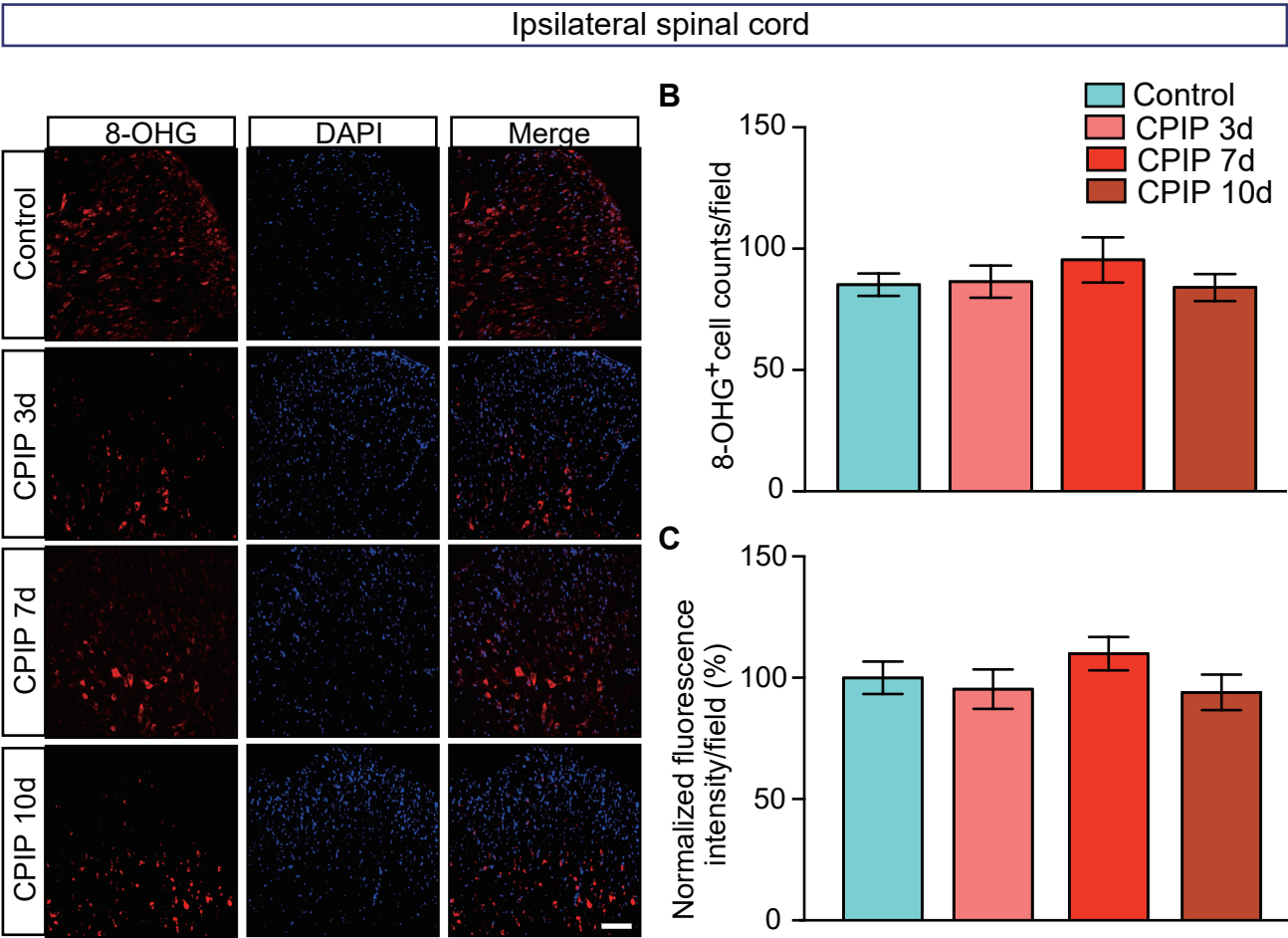

Supplement: Supplementary Materials — The supplementary materials contain the following figures and tables in one file: Suppl. Figure 1: original Western blot images. Suppl. Figure 2: high-quality hindpaw tissue RNA obtained for RNA-Seq. Suppl. Figure 3: oxidative stress-induced cellular damage is not present in ipsilateral spinal cord dorsal horn of CPIP model rats. Suppl. Figure 4: evaluation of oxidative stress status in female CPIP model rats. Suppl. Figure 5: persistent EA treatment reduces overactivation of glial cells in SCDH of CPIP model rats. Suppl. Figure 6: persistent EA or NAC treatment reduces proinflammatory cytokine overexpression in hindpaw tissues of CPIP model rats. Suppl. Table 1: sequence of primers used for qPCR. Suppl. Table 2: complete list of statistical results (mean, SEM, SD, and confidence interval). Suppl. Table 3: expression changes of genes involved in oxidative stress, antioxidant defense, and reactive oxygen metabolism process. [file 8035109.f1.zip › Suppl. Fig3 SCDH 8-OHG reduced.pdf]

Ipsilateral hind paw tissue of female rats

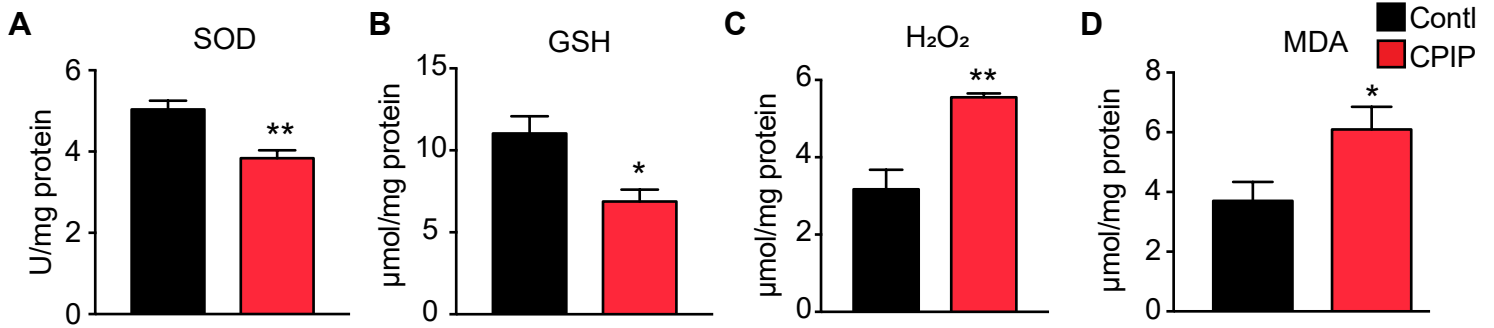

Ipsilateral spinal cord tissue of female rats

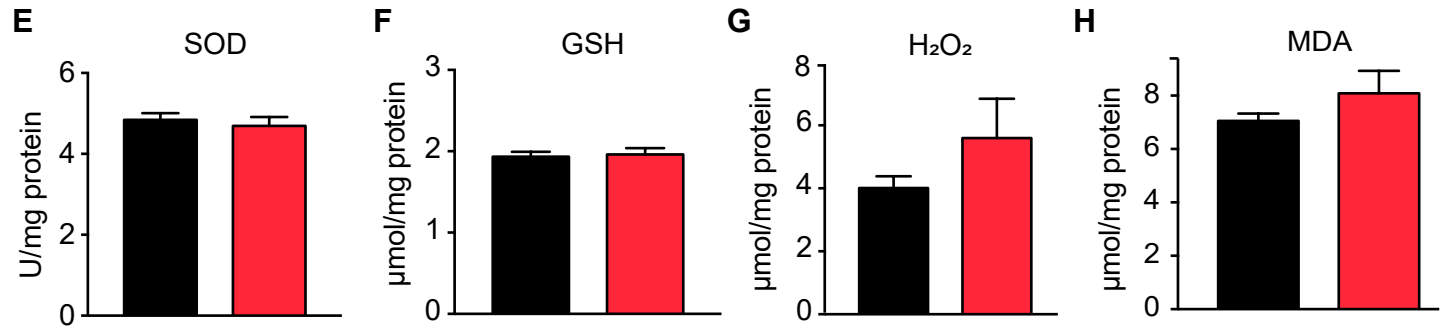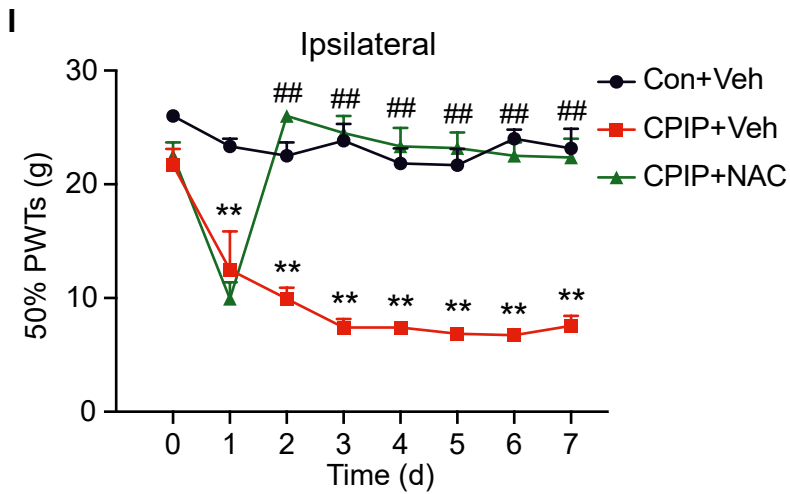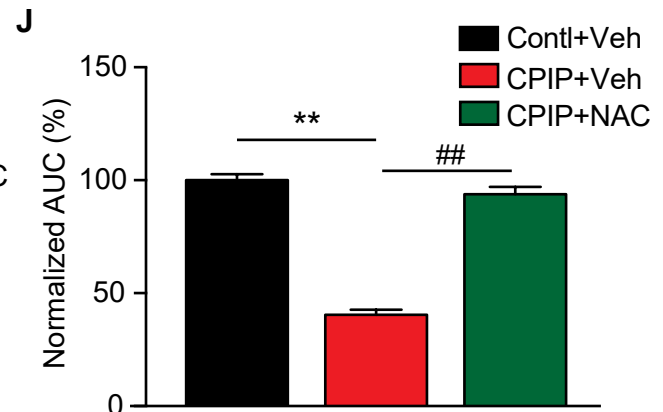

Supplement: Supplementary Materials — The supplementary materials contain the following figures and tables in one file: Suppl. Figure 1: original Western blot images. Suppl. Figure 2: high-quality hindpaw tissue RNA obtained for RNA-Seq. Suppl. Figure 3: oxidative stress-induced cellular damage is not present in ipsilateral spinal cord dorsal horn of CPIP model rats. Suppl. Figure 4: evaluation of oxidative stress status in female CPIP model rats. Suppl. Figure 5: persistent EA treatment reduces overactivation of glial cells in SCDH of CPIP model rats. Suppl. Figure 6: persistent EA or NAC treatment reduces proinflammatory cytokine overexpression in hindpaw tissues of CPIP model rats. Suppl. Table 1: sequence of primers used for qPCR. Suppl. Table 2: complete list of statistical results (mean, SEM, SD, and confidence interval). Suppl. Table 3: expression changes of genes involved in oxidative stress, antioxidant defense, and reactive oxygen metabolism process. [file 8035109.f1.zip › Suppl. Fig4 __PWT_ros.pdf]

**Suppl. Fig. 5**

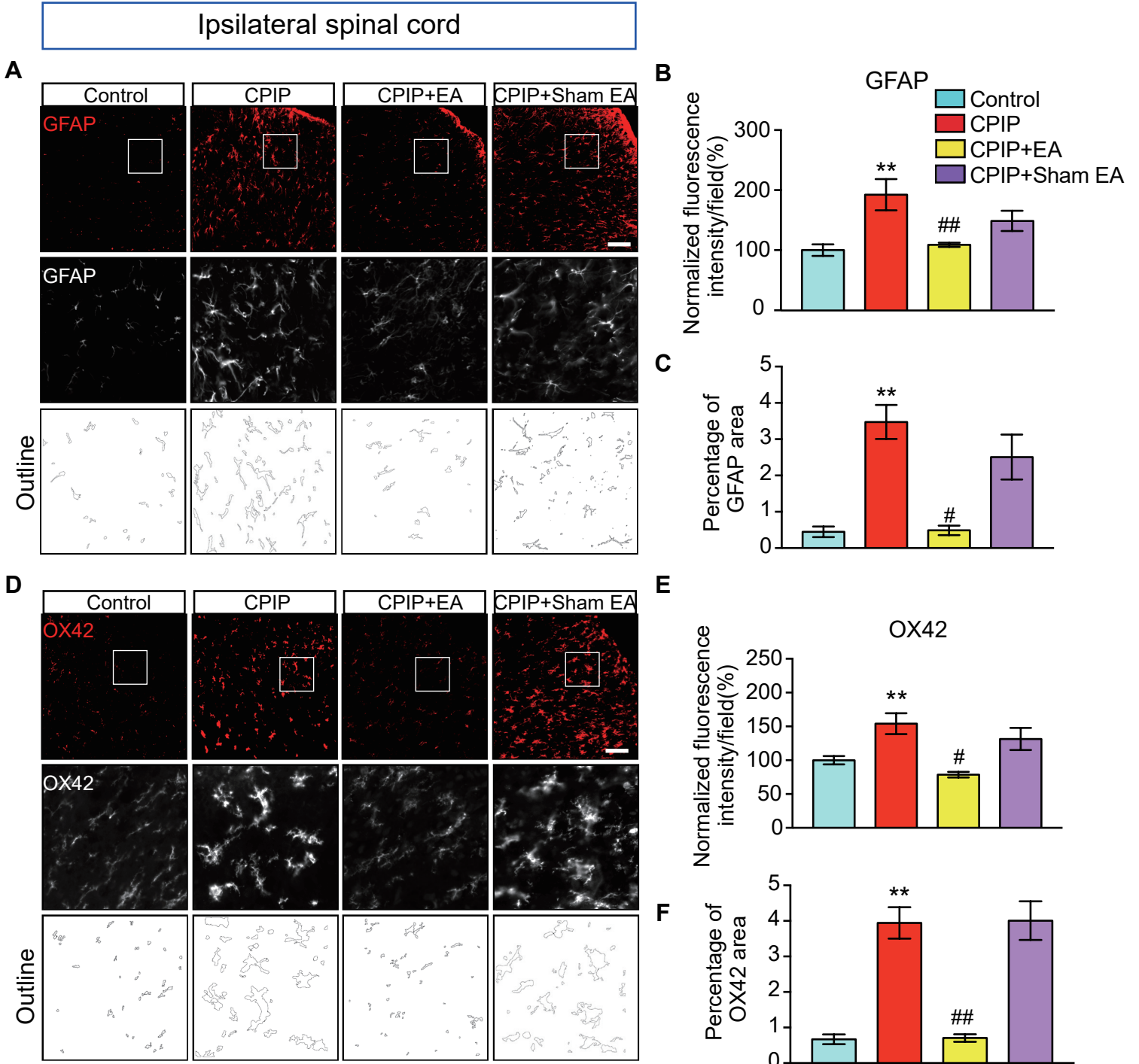

Supplement: Supplementary Materials — The supplementary materials contain the following figures and tables in one file: Suppl. Figure 1: original Western blot images. Suppl. Figure 2: high-quality hindpaw tissue RNA obtained for RNA-Seq. Suppl. Figure 3: oxidative stress-induced cellular damage is not present in ipsilateral spinal cord dorsal horn of CPIP model rats. Suppl. Figure 4: evaluation of oxidative stress status in female CPIP model rats. Suppl. Figure 5: persistent EA treatment reduces overactivation of glial cells in SCDH of CPIP model rats. Suppl. Figure 6: persistent EA or NAC treatment reduces proinflammatory cytokine overexpression in hindpaw tissues of CPIP model rats. Suppl. Table 1: sequence of primers used for qPCR. Suppl. Table 2: complete list of statistical results (mean, SEM, SD, and confidence interval). Suppl. Table 3: expression changes of genes involved in oxidative stress, antioxidant defense, and reactive oxygen metabolism process. [file 8035109.f1.zip › Suppl. Fig5 EA GFAP_OX42 reduced.pdf]

Suppl. Fig. 6

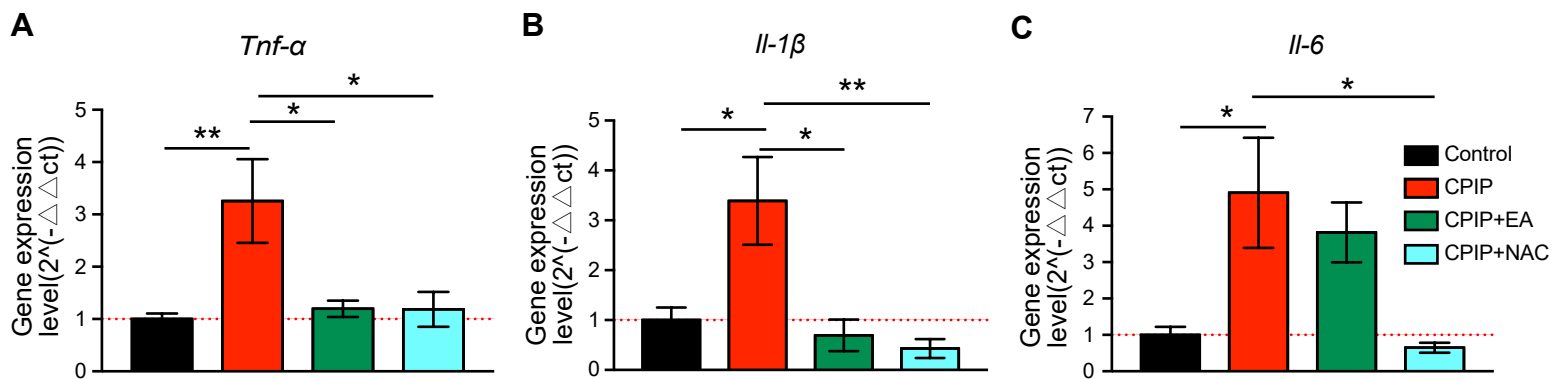

Supplement: Supplementary Materials — The supplementary materials contain the following figures and tables in one file: Suppl. Figure 1: original Western blot images. Suppl. Figure 2: high-quality hindpaw tissue RNA obtained for RNA-Seq. Suppl. Figure 3: oxidative stress-induced cellular damage is not present in ipsilateral spinal cord dorsal horn of CPIP model rats. Suppl. Figure 4: evaluation of oxidative stress status in female CPIP model rats. Suppl. Figure 5: persistent EA treatment reduces overactivation of glial cells in SCDH of CPIP model rats. Suppl. Figure 6: persistent EA or NAC treatment reduces proinflammatory cytokine overexpression in hindpaw tissues of CPIP model rats. Suppl. Table 1: sequence of primers used for qPCR. Suppl. Table 2: complete list of statistical results (mean, SEM, SD, and confidence interval). Suppl. Table 3: expression changes of genes involved in oxidative stress, antioxidant defense, and reactive oxygen metabolism process. [file 8035109.f1.zip › Suppl. Fig6 __.pdf]
